# Radial glial cells play a key role in echinoderm neural regeneration

**DOI:** 10.1186/1741-7007-11-49

**Published:** 2013-04-18

**Authors:** Vladimir S Mashanov, Olga R Zueva, José E García-Arrarás

**Affiliations:** 1 Department of Biology, University of Puerto Rico, PO Box 70377, San Juan, PR, 00936-8377, USA

**Keywords:** Cellular mechanisms, Central nervous system, Echinodermata, Injury, Neurogenesis, Radial glia, Regeneration, Sea cucumber

## Abstract

**Background:**

Unlike the mammalian central nervous system (CNS), the CNS of echinoderms is capable of fast and efficient regeneration following injury and constitutes one of the most promising model systems that can provide important insights into evolution of the cellular and molecular events involved in neural repair in deuterostomes. So far, the cellular mechanisms of neural regeneration in echinoderm remained obscure. In this study we show that radial glial cells are the main source of new cells in the regenerating radial nerve cord in these animals.

**Results:**

We demonstrate that radial glial cells of the sea cucumber *Holothuria glaberrima* react to injury by dedifferentiation. Both glia and neurons undergo programmed cell death in the lesioned CNS, but it is the dedifferentiated glial subpopulation in the vicinity of the injury that accounts for the vast majority of cell divisions. Glial outgrowth leads to formation of a tubular scaffold at the growing tip, which is later populated by neural elements. Most importantly, radial glial cells themselves give rise to new neurons. At least some of the newly produced neurons survive for more than 4 months and express neuronal markers typical of the mature echinoderm CNS.

**Conclusions:**

A hypothesis is formulated that CNS regeneration via activation of radial glial cells may represent a common capacity of the Deuterostomia, which is not invoked spontaneously in higher vertebrates, whose adult CNS does not retain radial glial cells. Potential implications for biomedical research aimed at finding the cure for human CNS injuries are discussed.

## Background

The recent decades have seen an overturn of two major dogmas in neurobiology. First, it is now accepted that the central nervous system (CNS) can continue producing new cells after an animal reaches adulthood. Second, it is becoming increasingly clear that glial cells, besides performing auxiliary functions, have also other, more active, roles, one of which is involvement of glia in adult neurogenesis, either as stem cells per se or as “niche cells”, which regulate mitotic activity, fate decisions, and differentiation of the progenitor cells [1-3]. Continuous generation of new neurons has been reported to occur in the CNS of a variety of invertebrate and vertebrate taxa, including human [4-8]. In some animals, adult neurogenesis not only results in production of new cells to support certain behaviors, sensory functions, or continuous lifelong body growth under normal physiological conditions, but also involves the ability to regenerate lesioned CNS regions. Within the vertebrate lineage, the highest regenerative capacity is seen in the CNS of fish and amphibians, whereas the least efficient regeneration is a characteristic of the mammalian CNS [6,9-11]. Therefore, there have been a growing number of studies of neural regeneration in regeneration-competent non-mammalian vertebrates aimed at getting insight into if and how regenerative response in the human nervous system can be improved.

Although factors that suppress mammalian neural regeneration have been identified, and studies on fish and amphibians have significantly contributed to our understanding of the fundamental mechanisms of CNS repair, this body of experimental work has not yet translated into a meaningful therapeutic effect [12]. In order to understand, which and how phylogenetically conserved latent mechanisms may be activated to their full potential to harness complete CNS regeneration in mammals, an extensive comparative analysis of animals with high regenerative capacities from phylogenetically relevant taxa should be carried out. From this perspective, the use of regeneration-competent non-mammalian vertebrates is completely justified, but not without certain drawbacks. First, at the neurohistological level, the CNS of all chordates shows complex architecture and diversity of glial cell types, the key players in neurogenesis [3], which complicates in vivo studies. Even the basal chordate, amphioxus, possesses multiple forms of glial cells [13]. The field would, therefore, benefit from experiments on model organisms with a simple CNS, which nevertheless should share basic design principles with the chordate CNS. Second, evidence is beginning to emerge that at least some components of the regenerative response in regeneration-competent vertebrates might have evolved locally at a taxon-specific level and have no counterparts in other animal groups [14]. Thus, an appropriate outgroup should be used in order to distinguish such evolutionary novelties from the most fundamental phylogenetically conserved mechanisms behind CNS regeneration.

Among available non-chordate CNS regeneration models, echinoderms arguably represent the best match to the above two criteria. Modern molecular systematics places the phylum Echinodermata (along with hemichordates) as a sister group to chordates within the monophyletic taxon Deuterostomia [15-17] making echinoderms a suitable group to inform biomedical research. Radial symmetry of the body plan has long been considered a somewhat confusing aspect of echinoderm biology, but nowadays it is becoming much less of a problem due to the growing appreciation that each of the radial nerve cords in an extant echinoderm is homologous to the dorsal nerve cord in chordates [18-23]. In spite of a relatively simple architecture, the echinoderm CNS shares fundamental principles of organization with the chordate CNS. One of the most important common features is the presence of radial glia. In the echinoderm CNS, radial glia is the only major glial cell type. These cells are similar to the chordate radial glia in a number of morphological and immunocytochemical characteristics [19,21,22,24,25] and, most importantly, also retain capacity of cell division in adult individuals, suggesting that they can be possibly involved in adult neurogenesis in these marine invertebrate deuterostomes [22].

Our previous research [25,26] has demonstrated that the radial nerve cord of adult sea cucumbers is capable of fast and efficient regrowth and re-connection after complete transection. Detailed microscopic analysis resulted in creation of an atlas of histology and ultrastructure of the normal and regenerating sea cucumber CNS and showed that the response to injury involved activation, dedifferentiation, and increased proliferation of glial cells [25]. However, the absence of cell type-specific markers precluded further studies. In this paper, we take an advantage of recently generated monoclonal antibodies [22] to provide the first comprehensive analysis of the role of radial glial cells in CNS regeneration in echinoderms. We now show that both glia and neurons undergo programmed cell death in response to injury, but it is the radial glia that take the leading role in subsequent regeneration by making up the leading tip of the growing regenerate, producing new cells through cell division and giving rise to new neurons. The newborn neurons persist long-term in the newly created segment of the radial nerve cord, suggesting their functional integration into the CNS circuitry.

## Results

### Organization of the uninjured radial nerve

**Figure 1:**
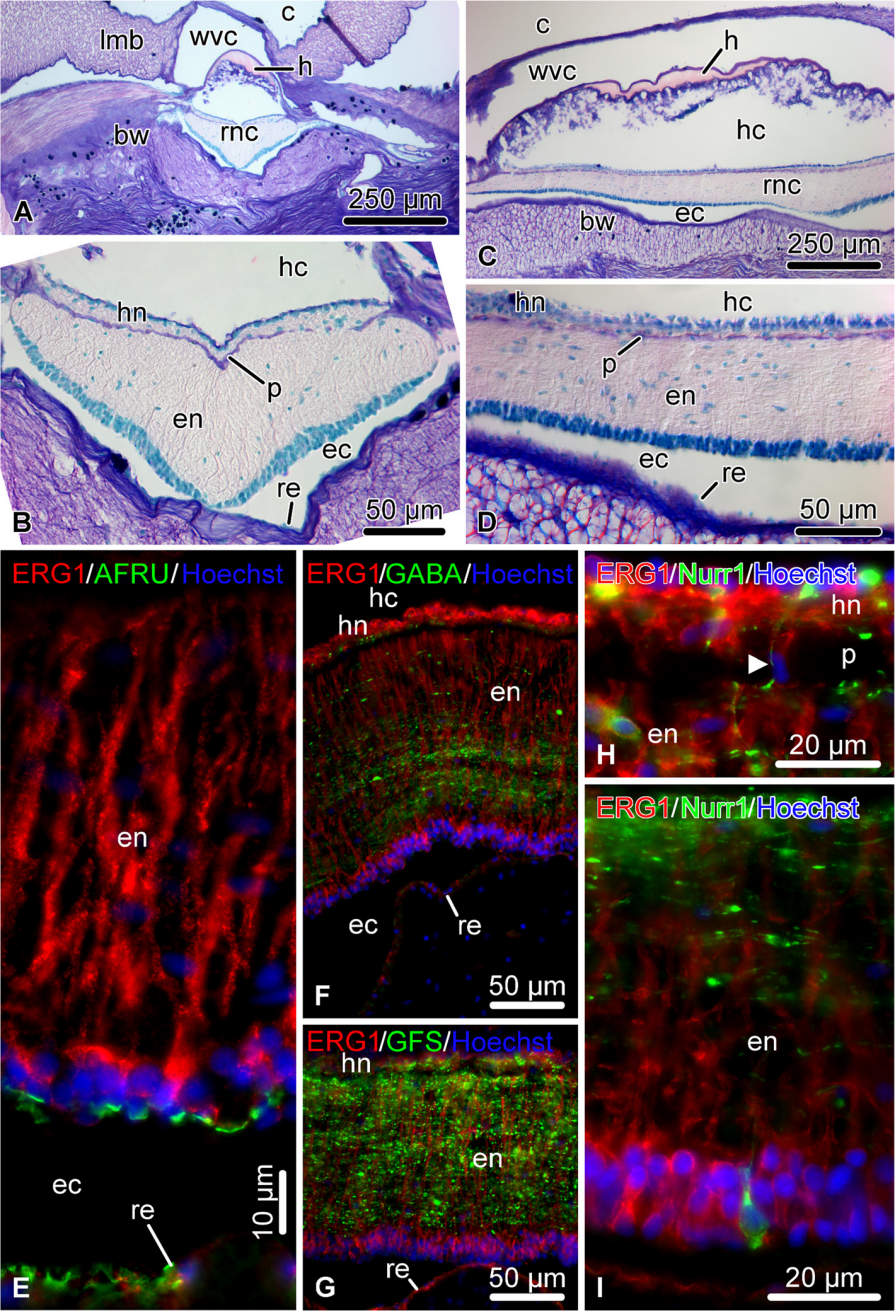
**Organization of the uninjured RNC.** (**A** – **D**) Microscopic anatomy of the RNC shown on transverse (**A, B**) and longitudinal (**C, D**) paraffin sections, Giemsa staining. (**B**) and (**D**) show higher magnification views of the RNC in (**A**) and (**C**), respectively. (**E** – **I**) Representative micrographs showing double immunolabeling of the RNC with the echinoderm radial glia-specific antibody ERG1 [22] and other glial and neuronal markers; cryosections. (**E**) ERG1 and AFRU (a rabbit polyclonal antiserum recognizing Reissner’s substance [30]), transverse section. (**F**) ERG1 and anti-GABA antibodies, longitudinal section. (**G**) ERG1 and anti-GFSKLYFamide [31] antibodies, longitudinal section. (**I, H**) ERG1 and anti-Nurr1 antibodies in the hyponeural (**H**) and ectoneural (**I**) neuroepithelia, longitudinal sections. Note a short bridge connecting the ectoneural and hyponeural neuroepithelia marked by an arrowhead in (**H**). bw, body wall connective tissue; c, coelom; ec, epineural canal; en, ectoneural neuroepithelium; h, hemal lacuna; hc, hyponeural canal; hn, hyponeural neuroepithelium; lmb, longitudinal muscle band; p, thin connective tissue partition separating the ectoneural and hyponeural neuroepithelia; re, roof epithelium; rnc, radial nerve cord; wvc, water-vascular canal.

Here, we provide a brief background on the organization of the sea cucumber central nervous system. For further details the reader is referred to Hyman [27], Heinzeller and Welsh [18], and Mashanov et al. [19,21,22]. Echinoderms possess a pentaradial body plan. Each of the five sections of their adult body is supplied with its own radial nerve cord (RNC), and all five radial nerves are joined together by the peripharyngeal nerve ring at the oral side of the body to form a single anatomical entity. The RNCs of sea cucumbers are immersed into the inner layer of the body wall connective tissue and are accompanied by other radial organs, such as the hemal lacuna, radial canal of the water-vascular system, and the longitudinal muscle band (Figure 1A-D). Each RNC consists of two adjacent parallel bands of nervous tissue, the thicker outer ectoneural neuroepithelium and the thinner inner hyponeural neuroepithelium, separated by a thin layer of connective tissue (Figure 1B, D). Each of these two bands is overlaid by a space, called the epineural and hyponeural canal, respectively, that runs longitudinally throughout the length of the cord. The outer walls of these canals are lined with a simple roof epithelium, which is made up of flattened glial cells and contains no neuronal elements (Figure 1B, D). The ectoneural and hyponeural neuroepithelia are composed of a dense supporting framework of radial glial cells with neuronal elements interspersed in the spaces between the cell bodies and processes of the glia (Figure 1E – I). The two neuroepithelia are connected by short bridges, which traverse the connective tissue partition and contain both glial and neuronal elements (Figure 1H). The radial glial cells show typical morphology. They are very tall and slender and arranged perpendicular to the plane of the neuroepithelium. Their cell bodies are mostly localized to the apical region of the neuroepithelium and give rise to a long occasionally branching basal process, which penetrates the whole thickness of the neural parenchyma and anchors to the basal lamina. In the uninjured nervous system, the radial glial cells of the ectoneural neuroepithelium and the flattened glial cells of the roof of the epineural canal produce and apically secrete a material, which shows positive immunoreactivity with antibodies against Reissner’s substance (RS) [21] (Figure 1E). In vertebrates, RS is produced by radial glia of the floor plate in embryogenesis and by secretory glia of the subcomissural organ in the adult brain [28,29].

### Regeneration of the radial nerve cord

As has been noted elsewhere [25], although the sequence of events that unfolds after a given type of injury is highly stereotyped in sea cucumbers, the pace of regeneration is often not exactly the same in different individuals resulting in differences in regeneration progress at any given time point. Therefore, here we use a staging system, which provides timing information, but is more accurately based on easily identifiable anatomical and histological criteria.

### Early post-injury phase (days 1 – 2)

**Figure 2:**
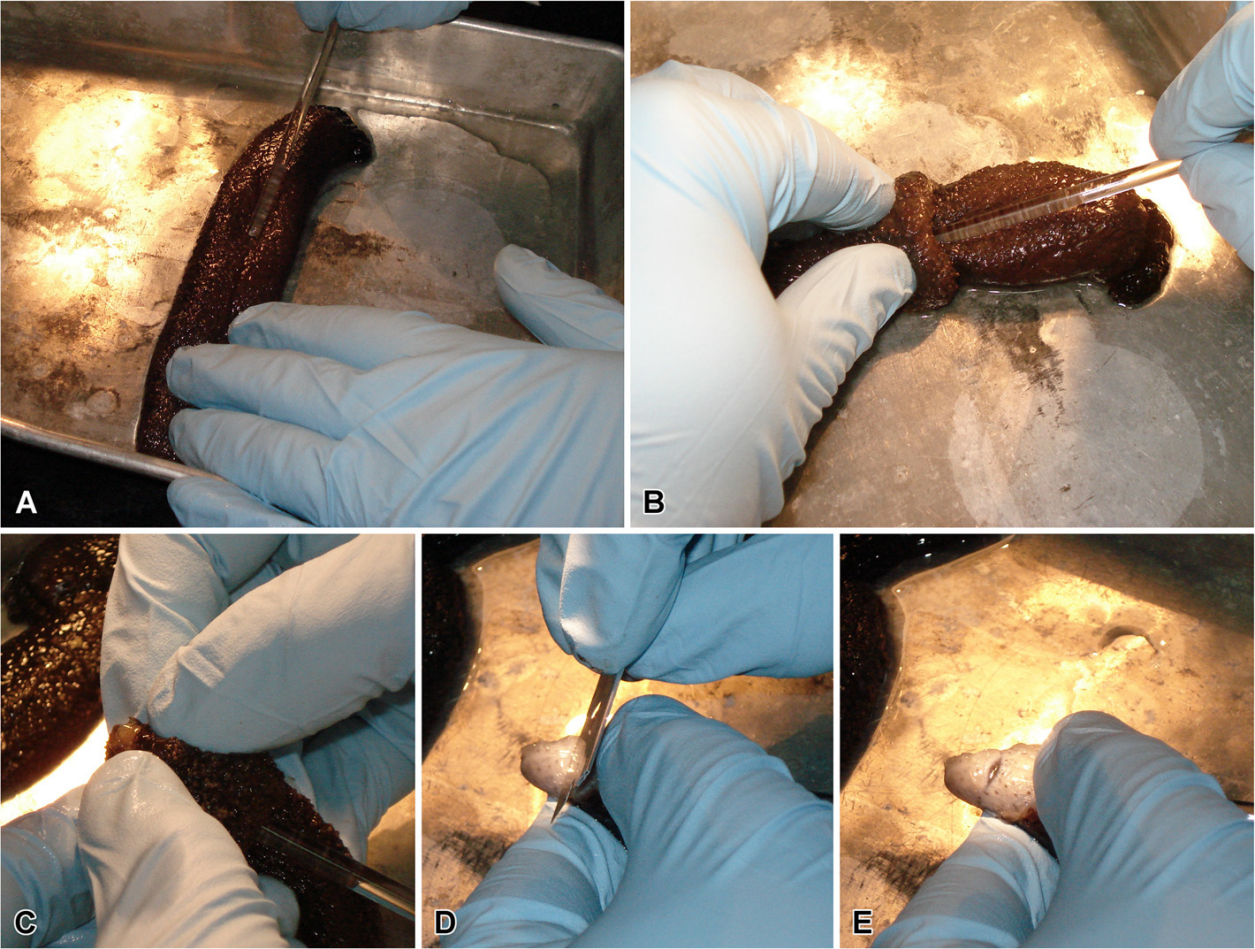
**The surgical procedure.** (**A**) The eviscerated animals were anesthetized to the point when they stopped responding to touch. (**B**, **C**) The inner layer of the body wall was exposed through the cloaca using a glass rod. (**D**, **E**) Using a razor blade, the mid-ventral radial organ complex, including the longitudinal muscle, water-vascular canal, and radial nerve cord, was cut at about the mid-body level without damaging the outer connective tissue layer and the epidermis.

**Figure 3:**
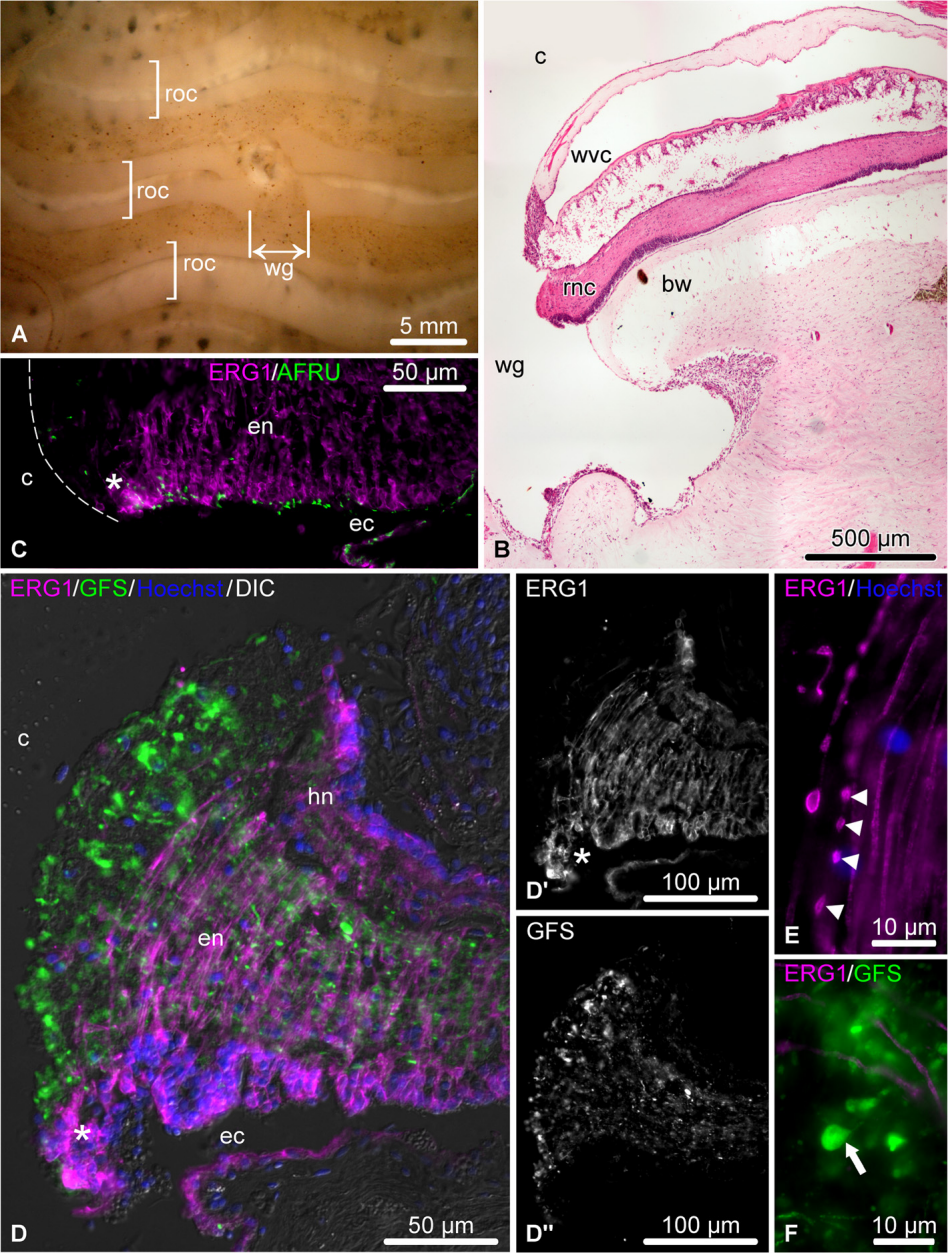
**Organization of the injured RNC during the early post-injury phase (days 1 – 2).** (**A**) Lesioned radial organ complex as viewed from the coelomic side of the body wall. (**B – F**) Longitudinal sections through the RNC. (**B**) Low-magnification view of the wound region; hematoxylin and esosin staining. (**C**) In the vicinity of the wound, the radial glial cells (magenta) still retain RS-like immunoreactivity (green) in the apical region of the ectoneural neuroepithelium. The dashed line shows the outlines of the cut end of the RNC. (**D**) Double labeling with the glial marker ERG1 (magenta) and the neuronal marker anti-GFSKLYFamide antiserum (green). (**D’**) and (**D”**) show these two types of labeling in separate channels. (**E**) Fragmentation (arrowheads) of glial processes (magenta) in the vicinity of the wound. (**F**) Neuronal processes (green) in injury area exhibit bulbous terminal swelling (arrow). bw, body wall connective tissue; c, coelom; ec, epineural canal; en, ectoneural neuroepithelium; hn, hyponeural neuroepithelium; rnc, radial nerve cord; roc, radial organ complex; wg, wound gap; wvc, water-vascular canal. Asterisk in (**C**, **D**, **D’**) indicates the zone of dedifferentiation in the ectoneural neuroepithelium with the radial glial cells preserving epithelial organization.

In our injury paradigm, one of the RNCs was cut at about the mid-body level (Figure 2, see Methods for details). The injury induces contraction of the longitudinal muscles and deformation of the underlying connective tissue of the body wall thereby creating a wide gap (~4 mm) between the cut ends of the radial organs (Figure 3A, B) and often making the stumps of the wounded radial nerve cord to protrude into the coelomic cavity (Figure 3B, C, D). Soon after injury, the ERG1-positive glial cells of both the ectoneural and hyponeural neuroepithelia undergo dedifferentiation at the wound surface. They lose their basal processes through fragmentation (Figure 3E), but their cell bodies maintain epithelial organization in the apical region of the neuroepithelium (Figure 3C, D, D’), and they still retain RS-like immunoreactivity at their apical surface (Figure 3C). Severed neuronal processes develop conspicuous bulbous terminal swellings (Figure 3D, D”, F). The dedifferentiation zone spreads along the RNC beyond the plane of initial injury, albeit only for a relatively short distance (~50-80 μm) (asterisk on Figure 3C, D, D’).

### Late post-injury (days 6 – 8)

**Figure 4:**
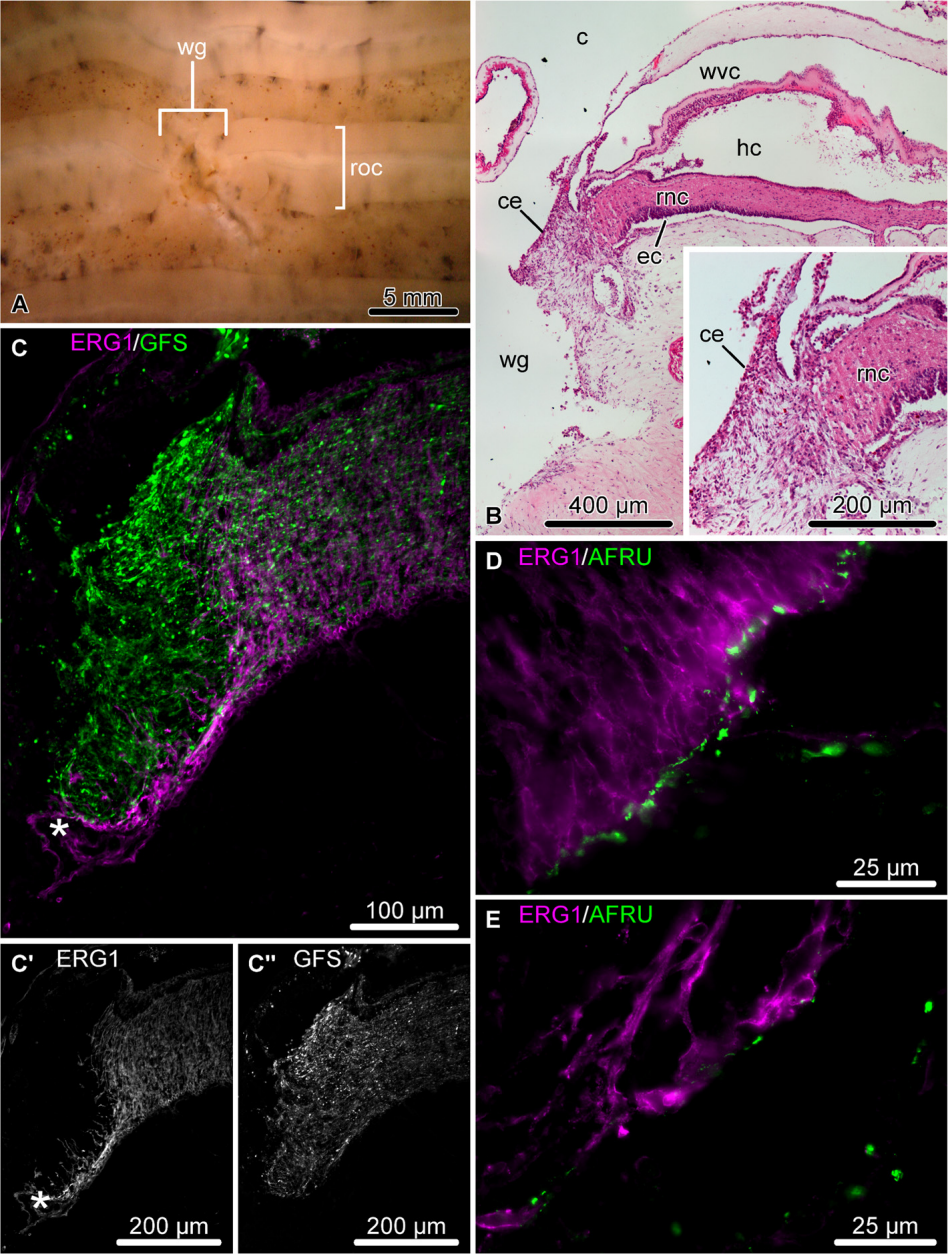
**Organization of the regenerating RNC during the late post-injury phase (days 6 – 8).** (**A**) Regenerating radial organ complex as viewed from the coelomic side of the body wall. (**B – E**) Longitudinal sections through the RNC. (**B**) Low-magnification view of the wound region; hematoxylin and eosin staining. The inset shows a detailed view of the distal tip of the RNC. (**C**) Double labeling with the glial marker ERG1 (magenta) and the neuronal marker anti-GFSKLYFamide antiserum (green). (**C’**) and (**C”**) show these two types of labeling in separate channels. Note the extended zone of glial dedifferentiation and a terminal swelling of the epineural canal (asterisk). (**D**, **E**) Double immunolabeling with the ERG1 (magenta) and anti-RS AFRU (green) antibodies. Note that the dedifferentiated radial glial cells at the distal tip of the RNC (**E**) produce less RS-like material, than do the glial cells in the more proximal regions (**D**). c, coelom; ce, coelomic epithelium; ec, epineural canal; hc, hyponeural canal; rnc, radial nerve cord; roc, radial organ complex; wg, wound gap; wvc, water-vascular canal.

By this stage, the wide wound gap still persists at the site of the transection (Figure 4A), but the injury surface of the radial nerve cord becomes partially or completely separated from the lumen of the main body cavity by the coelomic epithelium that migrates over the injury site and seals the wound (Figure 4B). Dedifferentiation of the RNC spreads much further from the plane of injury and affects deeper regions of the neuroepithelium (up to 300 μm) (Figure 4C – C”). The dedifferentiated glial cells at the cut end seal the epineural and hyponeural canals, and, in some individuals, form a swollen terminal ampulla (asterisk in Figure 4C, C’). They express significantly less RS-like material in their apical region than do the cells of the more distant regions of the RNC, which retain normal morphology (compare Figure 4D and E).

### Growth phase (days 8 – 12 post-injury)

**Figure 5:**
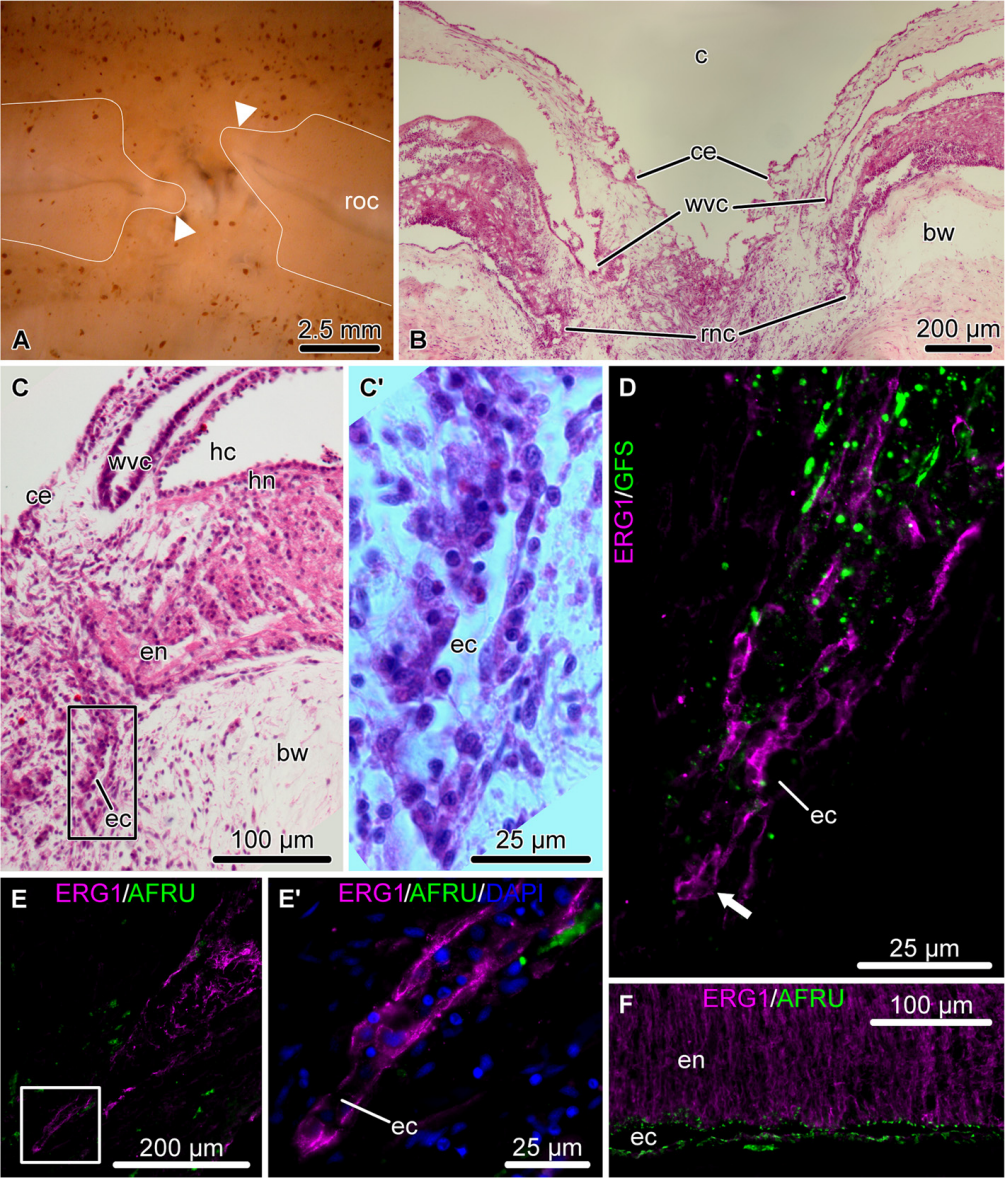
**Organization of the regenerating RNC during the growth phase (days 8 – 12 post-injury).** (**A**) Regenerating radial organ complex as viewed form the coelomic side of the body wall. The growing regenerates are labeled with arrowheads. Note that in this case the radial organs growing from the opposite sides of the wound are not aligned along the same axis. (**B – F**) Longitudinal sections through the regenerating RNC. (**B**) Low-magnification view of the regenerating radial organs; hematoxylin and eosin staining. (**C**) Detailed view of the growing tip of the RNC; hematoxylin and eosin staining. Note that the tip of the hyponeural cord grows slower than the ectoneural cord. (**C’**) shows higher magnification view of the boxed area in (**C**). (**D**) Growing ectoneural cord. Double labeling with the glial marker ERG1 (magenta) and the neuronal marker anti-GFSKLYFamide antiserum (green). Note that the leading tip (arrow) is made up of differentiated glial cells (magenta) and contains no neuronal elements (green). (**E**, **E’**, **F**) Double immunolabeling with the ERG1 (magenta) and anti-RS AFRU (green) antibodies. Note that the glial cells of the leading tip of the growing ectoneural cord do not produce RS-like material (**E**, **E’**), unlike the glial cells of the more proximal regions (**F**). (**E’**) shows a detailed view of the leading tip of the growing glial tube (boxed area in **E**). bw, connective tissue of the body wall; c, coelom; ce, coelimic epithelium; ec, epineural canal; en, ectoneural cord; hc, hyponeural canal; hn, hyponeural cord; rnc, radial nerve cord; roc, radial organ complex; wvc, water-vascular canal.

**Figure 6:**
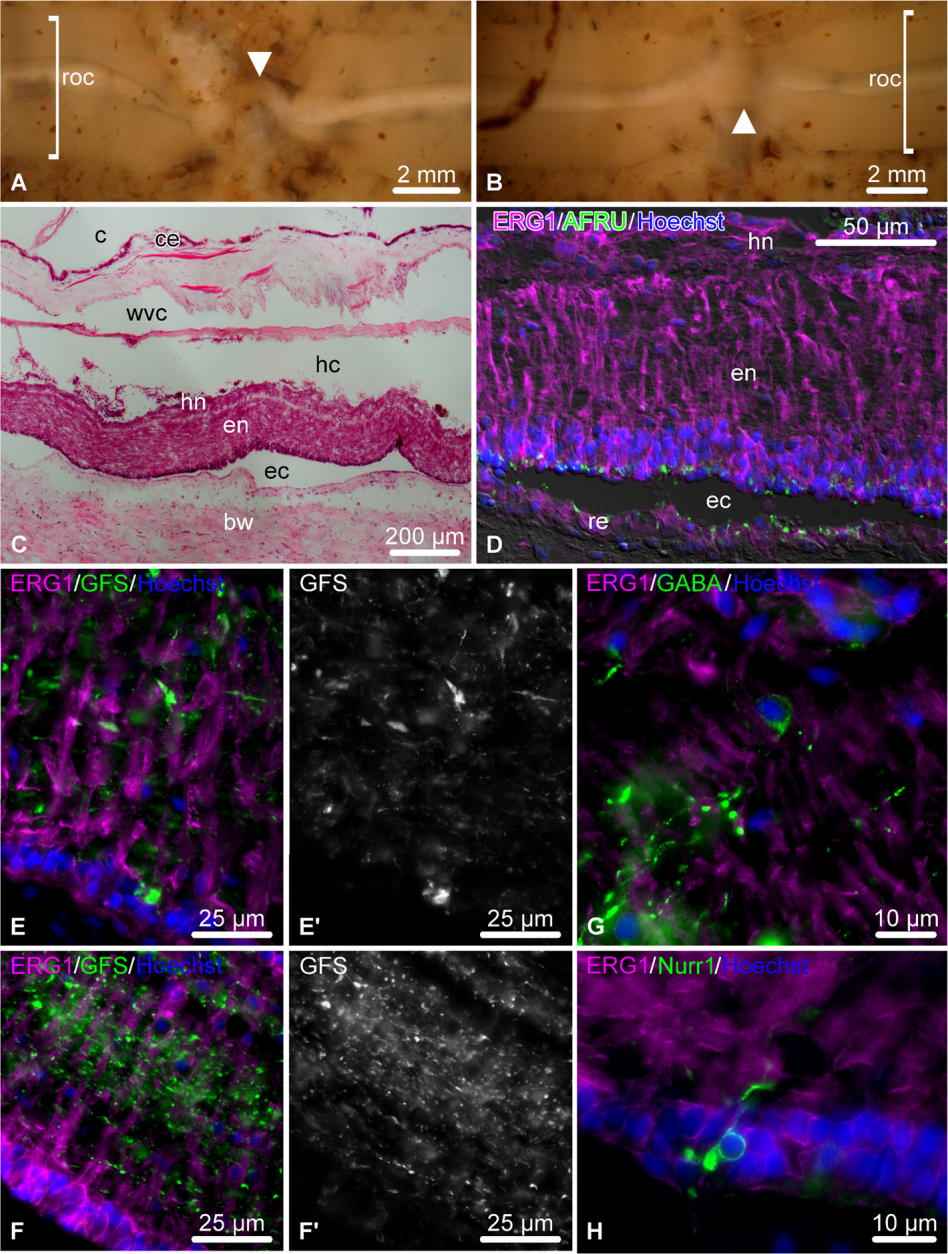
**Organization of the regenerating RNC during the late regenerate phase (21+ days after injury).** (**A**, **B**) Regenerating radial organ complex as viewed form the coelomic side of the body wall on day 28 and day 42 post-injury. Arrowheads indicate the regenerated radial organ complex bridging the wound gap. The shape of the regenerated structure in (**A**) suggests that the growing regenerates were not aligned along the same axis, but, nevertheless, were able to meet and fuse. (**C** – **H**) Longitudinal sections through the regenerated RNC. (**C**) General morphology of the regenerated radial organs on day 21 post-injury; hematoxylin and eosin staining. (**D**) Double immunolabeling of the RNC on day 21 post-injury with the ERG1 (magenta) and anti-RS AFRU (green) antibodies. Note that the radial glial cells of the newly regenerated segment of the RNC fully restored their palisade-like morphology and fully resumed their ability to produce and secrete the RS-like material. (**E** – **F’**) Double labeling with the glial marker ERG1 (magenta) and the neuronal marker anti-GFSKLYFamide antiserum (green) of the newly regenerated segment of the RNC (**E**, **E’**) and the region not affected by the injury (**F**, **F’**) on day 21 post-injury. (**E’**) and (**F’**) show the labeling with the anti-GFSKLYFamide antiserum in a separate channel. Note that the neuropil on day 21 has not yet completely restored its normal organization (compare with Figure 7). (**G**, **H**) Double labeling of the newly regenerated segment of the RNC with the glial marker ERG1 (magenta) and the anti-GABA (**G**) or the anti-Nurr1 antisera (**H**) (green) on day 21 post injury. bw, body wall connective tissue; c, coelom; ce, coelomic epithelium; ec, epineural canal; en, ectoneural neuroepithelium; hc, hyponeural canal; hn, hyponeural neuroepithelium; re, roof epithelium; roc, radial organ complex; wvc, water-vascular canal.

By days 8 – 12 after injury, the wound gap is still clearly discernible in the form of a deep furrow (Figure 5A, B). The coelomic epithelium of the body wall completely covers the wound surface, thereby separating the regenerating radial organs from the lumen of the coelomic cavity (Figure 5B, C). The two regenerates that have been developed on either side of the wound start growing towards each other across the wound gap beneath the coelomic epithelium (Figure 5A – C’). In some cases, the regenerates are laterally displaced and no longer aligned along the same axis (as shown on Figure 5A). In spite of this, in all animals examined at later time points the two regenerates meet and fuse together to restore the anatomical continuity (see below, Figure 6A).

At this stage, the dedifferentiated tips of the radial nerve stumps form thin tubular outgrowths penetrating into the connective tissue that fills the wound gap (Figure 5B-E’). It is important to note that the walls of the leading tip of the tubular outgrowths are formed almost exclusively of flattened dedifferentiated glial cells (Figure 5D,E’) with very few, if any, neuronal elements (Figure 5D). The glial cells at the growing tip stop producing and secreting RS-like material (Figure 5E, E’), whereas the glia the more proximal regions of the regenerate and of the uninjured regions of the radial nerve cord still show positive immunoreactivity (Figure 5F).

The ectoneural and hyponeural bands of the RNC form separate tubular rudiments that grow parallel to each other, however, the ectoneural band regenerates considerably faster (Figure 5С).

### Late regenerate (21+ days post-injury)

**Figure 7:**
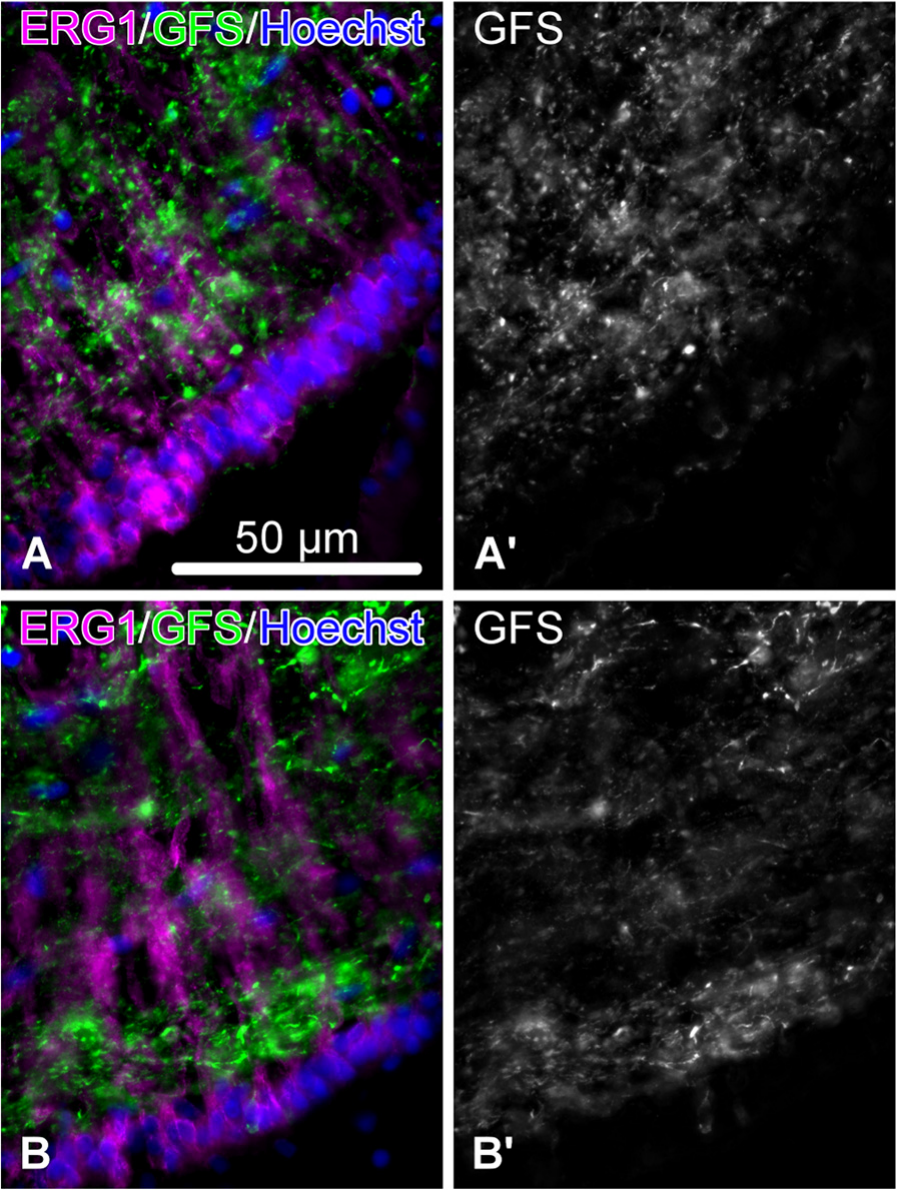
**Day 145 post injury.** Organization of the neuropil in the fully regenerated segment of the RNC (**A**, **A’**) and in the region of the RNC not affected by the injury (**B**, **B’**). Double labeling with the glial marker ERG1 (magenta) and the neuronal marker anti-GFSKLYFamide antiserum (green). (**A’**) and (**B’**) show the labeling with the anti-GFSKLYFamide antiserum in a separate channel.

This is the stage when the two growing regenerates meet to bridge the injury gap and restore the anatomical continuity. The newly regenerated segment of the radial organs then starts to gradually resume its normal appearance (Figure 6A – C). The radial glial population of the regenerated part of the radial nerve fully restores its palisade-like organization and is indistinguishable from the glia of the uninjured radial nerve (Figure 6D, E, F). These re-differentiated radial glial cells fully resume the ability to produce RS-like material, which accumulates in the apical region of the neuroepithelium, as in the uninjured animals (Figure 6D). The newly created region of the nerve cord is extensively populated with neuronal cell bodies and processes (Figure 6E, E’, G, H). On day 21 the nerve fibers in the regenerated segment of the RNC are not yet as orderly organized as in the regions not affected by the injury (compare Figure 6E, E’ and F, F’), but with time the neuropil completely resumes the normal morphology (Figure 7).

### Glial cells account for the majority of cell divisions in the normal and regenerating RNC

**Figure 8:**
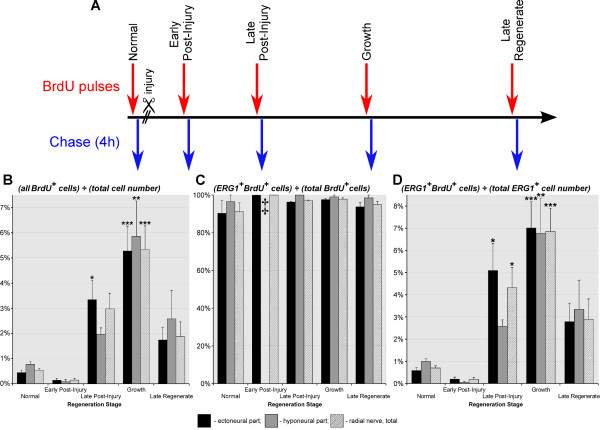
**Cell division dynamics in the normal and regenerating RNC.** (**A**) BrdU labeling paradigm employed to quantify cell division. (**B**) Diagram showing the percentage of all BrdU-incorporating cells (irrespective of their phenotype) normalized to the total number of cells in the tissue. (**C**) Diagram showing how many of the BrdU-incorporating cells are ERG1-positive glial cells. (**D**) Diagram showing how many of the ERG1-positive glial cells incorporate BrdU. Results are represented as mean (percentage) ± standard error. *P < 0.05, **P < 0.01, ***P < 0.001. ‡ indicates that the value is missing because BrdU-incorporating cells were absent in three of the four animals. The the ratio of the number of BrdU^+^ ERG1^+^ cells divided by the total number of BrdU^+^ cells is impossible to define in these animals (division of zero by zero). Therefore, neither mean value nor standard error were calculated. In the fourth animal, there were only two BrdU^+^ cells in the hyponeural neuroepithelium, one of them was BrdU^+^ ERG1^+^, whereas the other was BrdU^+^ ERG1^-^.

**Figure 9:**
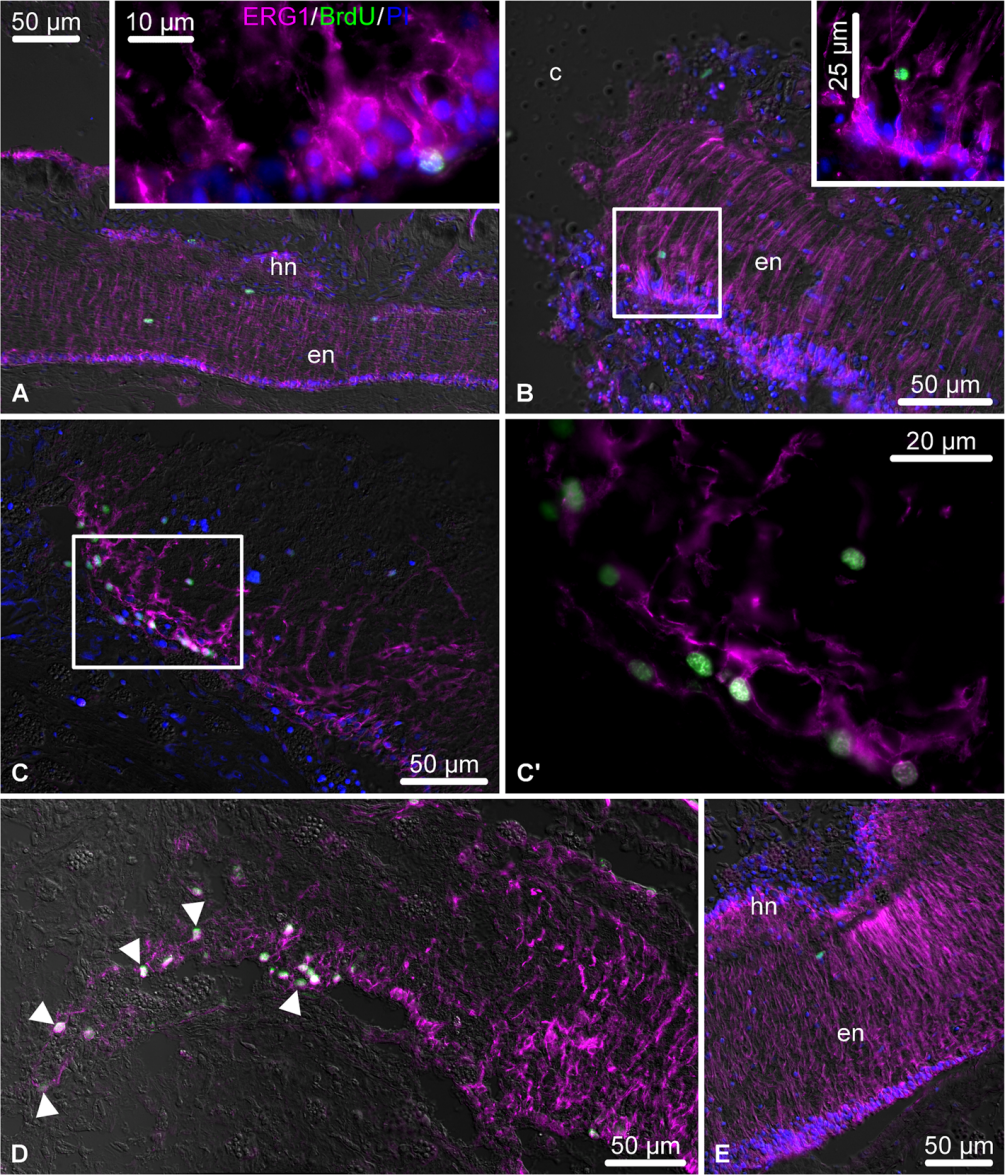
**Representative micrographs showing distribution of BrdU-incorporating cells (green) in the uninjured and regenerating RNC (single BrdU injection, 50 mg/kg, followed by a 4 h chase period).** The radial glial cells (magenta) are visualized by immunostaining with the ERG1 monoclonal antibody. Nuclei are stained with propidium iodide (PI, blue). All micrographs are longitudinal sections with the plane of injury/regenerate to the left. (**A**) The RNC of an uninjured animal. The ***inset*** shows a high maginification view of a BrdU-incorporating radial glial cell. (**B**) Early post-injury phase (day 1). The ***inset*** shows a higher magnification view of the boxed area. (**C**, **C’**) Late post-injury stage (day 6 post-injury). Note numerous BrdU-incorporating cells among the dedifferentiating radial glia. (**C’**) shows higher magnification of the boxed area in (**C**) (dedifferentiating region of the RNC). (**D**) Growth phase (day 8 post-injury). Note abundant BrdU-positive cells in the growing tubular glial regenerate (arrowheads). (**E**) Late regenerate (day 21 post-injury). en, ectoneural neuroepithelium; hn, hyponeural neuroepithelium.

In order to determine the relative contribution of glial cells to cell proliferation we combined BrdU pulse-chase technique (single pulse followed by a 4h chase period) (Figure 8A) with labeling with the specific glial marker ERG1. The cell counting data are shown in a graphical form in Figure 8B – D, the corresponding numerical values are listed in Additional file 1: Table S1, the results of ANOVA test are summarized in Additional file 2: Table S2, and Figure 9 shows representative micrographs used in the cell counting assays. As has been shown elsewhere [22], BrdU incorporation occurs at a certain basal level even in the uninjured sea cucumber central nervous system, suggesting that new cells are being continuously produced in the adult CNS of echinoderms. No statistically significant changes in cell division were observed at the early post-injury stage. At the late post-injury stage, the number of BrdU-incorporating cells starts to increase significantly in the ectoneural part of the RNC (~7.4-fold compared to the uninjured animals) (Figure 8B). During the growth phase, this increase affects both the ectoneural and hyponeural cords and the numbers reach the maximum values (~10-fold increase vs normal animals), before starting to return to the normal levels in the late regenerate. More importantly, even though the overall number of BrdU-positive cells increased significantly, the ratio of ERG1^+^ BrdU^+^ cells to the total number of BrdU^+^ cells, representing the contribution of glial cells to cell proliferation, remained essentially constant both in the uninjured and regenerating animals (Figure 8B, C, Additional file 1: Table S1 and Additional file 2: Table S2). The high value of this ratio (~ 91–100%) suggests that new cells in the normal and regenerating CNS of sea cucumbers are produced almost exclusively by ERG1^+^ radial glia. The dominant role of glial cells in cell division is also reflected by the fact that the temporal changes in proportion of the glial population involved in BrdU incorporation follow exactly the same pattern as the ratio of the total number of BrdU^+^ cells to the overall number of cells in the CNS (compare Figure 8B and D). It is important to note the difference in distribution of proliferating cells in the normal and regenerating animals. The BrdU^+^ cells are randomly scattered in the uninjured nervous system, but during the late-post injury and growth phases, BrdU-positive staining is mostly seen in the dedifferentiated glia at the distal tip of the regenerate (Figure 9C, D).

### Post-mitotic progeny of the radial glial cells gives rise to new neurons in radial nerve cord regeneration

**Figure 10:**
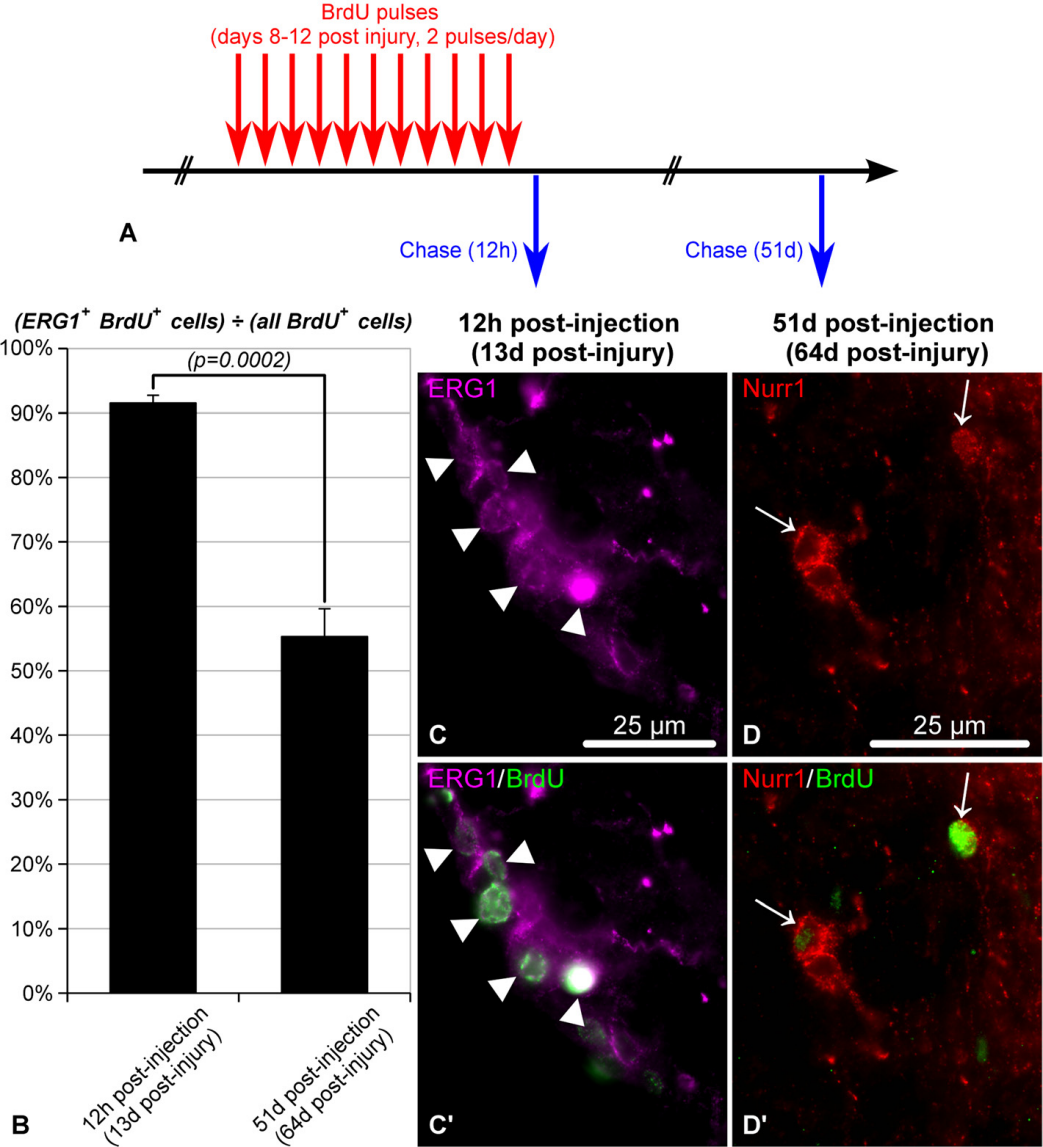
**Proliferating ERG1-positive glial cells give rise to neurons in the regenerating RNC.** (**A**) BrdU labeling paradigm employed to label proliferating glial cells and trace their progeny. Multiple BrdU injections (50 mg/kg, every 12 hours) were given during the growth phase of regeneration (days 8 thru 12 post-injury). The tissues were fixed at two time points: 12 hours and 51 days after the last BrdU injection (on day 13 and day 64 post-injury, respectively). (**B**) Proportion of ERG1-positive glial cells among BrdU-positive cells. Note that shortly after BrdU administration during the growth phase, the vast majority of BrdU-incorporating cells show ERG-positive glial phenotype. This number significantly decreases on day 51 after the last BrdU injection, when ERG1-negative cells (neurons) represent almost half of the BrdU-positive progeny. (**C**, **C’**) Representative micrographs showing BrdU-incorporating ERG1-positive radial glial cells (arrowheads) 12 h after the last BrdU injection. (**D**, **D’**) Representative micrographs showing Nurr1^+^ BrdU^+^ neurons (arrows) on day 51 after the last BrdU injection (day 64 post-injury).

We then asked what happens to those cells, which are produced during the peak of cell division at the growth stage of regeneration. We employed multiple BrdU injections (50 mg/kg, every 12 hours, see Methods and Figure 10A) to label dividing cells between day 8 and day 12 post-injury. Initially, after 4 days of BrdU saturation, the vast majority (~92%) of BrdU-labeled cells were positively stained with the glial marker ERG1 (Figure 10B – C’). However, 51 days later (day 63 post-injury), almost half (~45%) of the BrdU^+^ progeny no longer showed positive staining with ERG1 (Figure 10B) and at least some of them started expressing neuronal markers, such as Nurr1 (Figure 10D, D’) and GFSKLYFamide. Given that almost all glial cells in sea cucumbers are labeled with ERG1 and that the vast majority of ERG1-negative subpopulation in the central nervous system are morphologically identified as neurons [22], we conclude that part of the BrdU-labeled progeny of radial glial cells gives rise to neurons in CNS regeneration. Importantly, BrdU-labeled cells expressing neuronal markers were found as long as 133 days after the last BrdU injection (the last time point analyzed, not shown), suggesting that the newly generated neurons survive long term in the regenerated segment of the RNC.

### The peak of cell death in both neurons and glial cells occurs at the early post-injury stage

**Figure 11:**
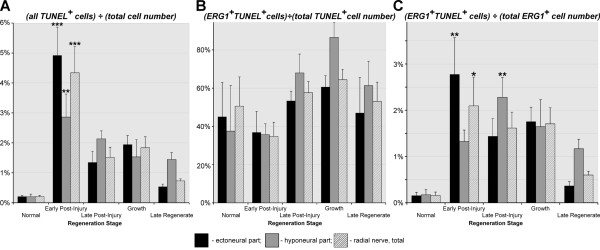
**Dynamics of programmed cell death as determined by TUNEL assay in the normal and regenerating RNC.** (**A**) Relative abundance of all TUNEL-positive cells (irrespective of their phenotype) normalized to the total number of cells in the tissue. (**B**) Proportion of TUNEL-positive ERG1^+^ glial cells in relation to the total number of TUNEL-positive cells. (**C**) Proportion of TUNEL-positive ERG1^+^ glial cells in relation to the total number of ERG1^+^ glial cells. Results are represented as mean (percentage) ± standard error. *P < 0.05, **P < 0.01, ***P < 0.001.

**Figure 12:**
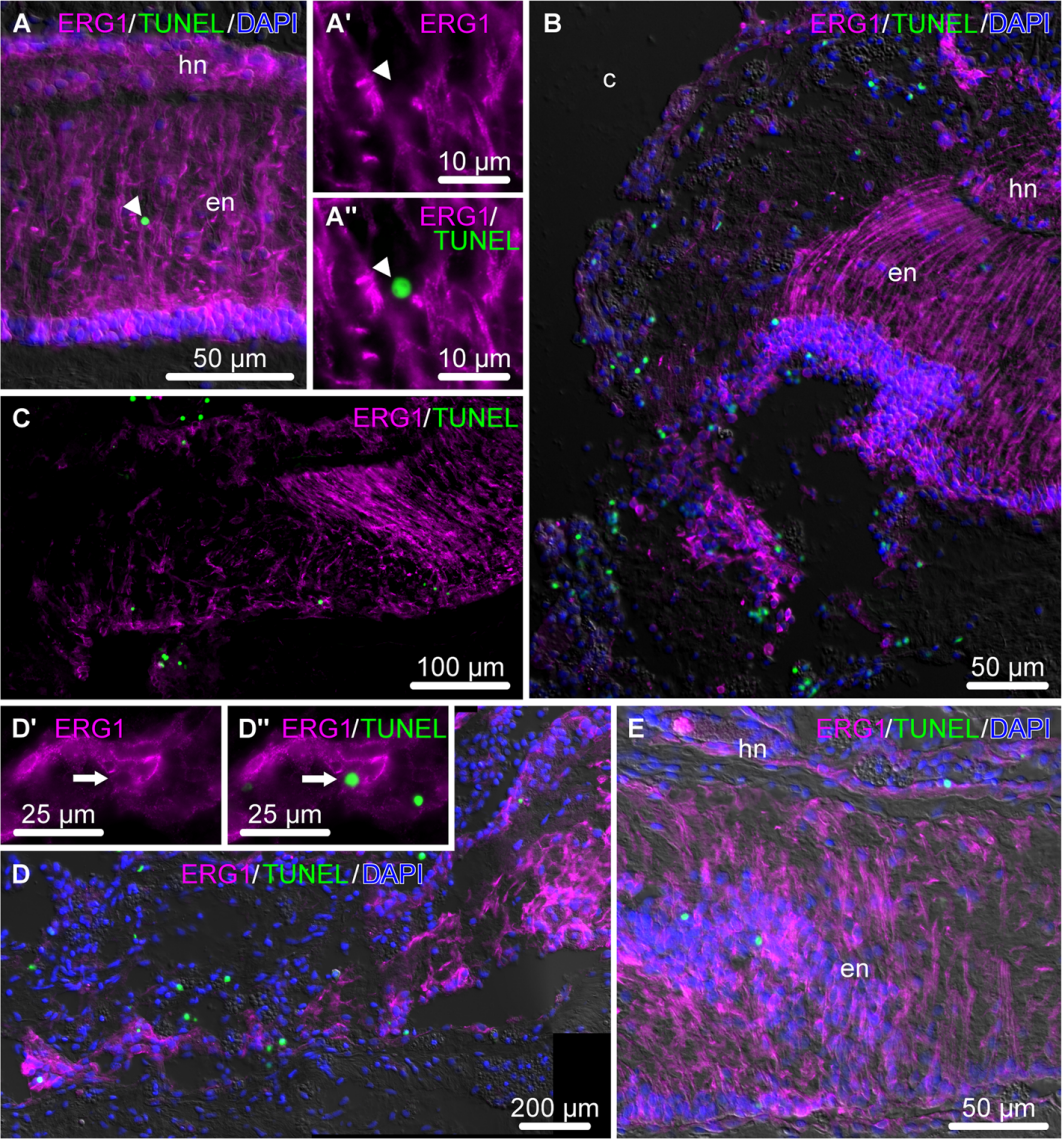
**Representative micrographs showing distribution of TUNEL-positive cells (green) in the uninjured and regenerating RNC.** The radial glial cells are stained with the ERG1 monoclonal antibody (magenta), and the nuclei are stained with DAPI (blue). All micrographs are longitudinal sections with the plane of injury or growing regenerate to the left. (**A**) Uninjured RNC. **A’** and **A”** show a high magnification view of the TUNEL-positive ERG1-negative cell marked with an arrowhead. (**B**) Abundant TUNEL-positive cells in the vicinity of the injury plane at the early post-injury stage (day 1). (**C**) Late post-injury stage (day 8). (**D**) Growing glial tubular rudiment on day 10 post-injury. **D’** and **D”** show a high magnification view of a TUNEL-positive ERG1-positive glial cell (marked with an arrow). (**E**) Late regenerate (day 21 post-injury).

We employed TUNEL (terminal deoxynucleotidyl transferase-mediated dUTP end labeling) assay to quantify the effect of induced programmed cell death on glial population of the RNC. These results are summarized in bar plots in Figure 11, the corresponding numerical values and results of ANOVA are shown in Additional file 3: Table S3 and Additional file 4: Table S4, respectively, and the representative micrographs are shown in Figure 12. Here, we confirm our previous observations [22] that continuous production of new cells in the uninjured adult echinoderm CNS (see above) is counter-balanced by a certain basal level (~0.2% of the total cell number) of programmed cell death. After injury, programmed cell death showed an opposite dynamics when compared to cell proliferation. The overall number of TUNEL-positive cells reached the maximum levels in the vicinity of the injury at the early post-injury stage (4.34%, corresponding to roughly a 20-fold increase compared to the uninjured animals), then started to return to the basal levels as regeneration proceeded (Figure 11A, Additional file 3: Table S3A). In contrast to cell division, cell death was found to equally affect both glial cells and neurons (Figure 11B, 12; Additional file 3: Table S3B), and the contribution of ERG^+^ glial cells to the total number of TUNEL^+^ cells, although showing some fluctuations, did not change significantly between the analyzed conditions (*p* > 0.087) (Figure 11B; Additional file 3: Table S3B and Additional file 4: Table S4). The percentage of glial population undergoing cell death reached the peak value at the early and late post-injury phases in the ectoneural and hyponeural parts of the RNC, respectively (Figure 11C, Additional file 3: Table S3C). During the growth phase, the proportion of TUNEL-positive glial cells still remains somewhat elevated (1.64% and 1.71%, corresponding to 9.6 and 10.7 fold in the ectoneural and hyponeural cords, respectively), although marginally insignificant (with p-values of 0.069 and 0.053, in the ectoneural and hyponeural parts of the RNC, respectively) compared to the normal nervous tissue. In the late regenerate, this ratio did not differ statistically from the values for the normal RNC.

## Discussion

This paper presents data indicating that radial glial cells play a leading role during CNS regeneration in echinoderms. Echinoderm radial glial cells share a number of key characteristics with the radial glia of chordates. These include (a) an orthogonal orientation relative to the plane of the neuroepithelium; (b) elongated shape allowing the cell to stretch between the apical and basal surfaces of the neuroepithelium; (c) apical cilia protruding into the lumen of the epineural/hyponeural canal (in cryptosyringid echinoderms) or central canal (in chordates); (d) conspicuous bundles of intermediate filaments in the cytoplasm; (e) ability to produce and secrete Reissner’s substance [19,21,22,24]. It has been proposed that the radial glia is a phylogenetically ancient cell type in the deuterostome CNS, whose origin might have predated the diversification of the Chordata and Ambulacraria (Echinodermata + Hemichordata) lineages within the clade Deuterostomia [21,22]. Although interesting, this hypothesis needs further testing in future studies, as it is not absolutely clear if the similarity between the radial glial cells of echinoderm and chordates is due to true homology or convergence imposed by developmental constraints.

Synthesis of the data produced in the present study with our previous ultrastructural analysis of the regenerating CNS in sea cucumbers [25] suggests that activation of radial glial cells is one of the defining components of the regenerative response. The glial cells do not show any signs of reactive gliosis. They undergo dedifferentiation by loosing their hallmark basal processes and also stop producing the Reissner’s substance. However, this dedifferentiation does not affect the epithelial nature of the cells. The dedifferentiated radial glial cells are organized into an epithelium, which seals the cut end of the stump and forms a tubular outgrowth with a central lumen continuous with that of the epineural or hyponeural canal. These cells then show a considerable increase in their cell division rate and the tubular glial rudiments grow towards each other into the lesion site from both the anterior and posterior stumps and eventually fuse together to bridge the injury gap. As the glial regenerates grow, the newly formed regions behind the leading tip start their re-differentiation and become re-populated with neurons, at least some of which derive from the radial glia. Thus, CNS regeneration in sea cucumbers not only involves radial glia-mediated bridging of the wound gap and axonal outgrowth, but also production of new neurons by actively proliferating radial glia cells, i.e., represents genuine post-traumatic neurogenesis. Importantly, the presence of cell division and even production of new nerve cells does not necessarily equals to neurogenesis in functional sense [6]. For example, many adult newborn neurons in vertebrates undergo programmed cell death within a few days or weeks, if they do not receive appropriate stimulation [6,32]. In case of post-traumatic neurogenesis in sea cucumbers, the newly generated neurons remained alive long term for as long as 133 days, expressed markers typical of adult sea cucumber neurons, and established synaptic connections (see Figure 12G in [25]), suggesting that they might have become successfully integrated into CNS circuitry. However, future behavioral and physiological experiments are needed to unequivocally test for complete functional restoration in the newly regenerated segment of the RNC. Thus, the efficient post-traumatic neurogenesis seen in echinoderms involves response of radial glial cells, including dedifferentiation, increased cell division and production of new presumably functional neurons.

In regeneration-competent non-mammalian vertebrates, the cellular events, which are triggered in response to CNS injury, are similar to what we present here for echinoderms. Although some signs of reactive gliosis can be initially seen immediately after injury (such as swelling of glial processes), this type of response eventually subsides, as the radial glia cells, which persist into adulthood in these animals, never form a glial scar. Instead, these cells seal the cut end of the stump and form glial outgrowths, which either grow caudally past the plane of amputation (in case of tail regeneration) or migrate into the lesion site and fill the injury gap (after spinal cord transection). Most importantly, the activated glial cells are engaged in post-traumatic neurogenesis through extensive cell division and production of new functional neurons [6,9-11,33].

In mammals, CNS injury triggers a cascade of events, which constitute extensive reactive gliosis [34-36]. Response to CNS insult involves proliferation of astrocytes in the vicinity of the injury, but this cell division does not lead to regeneration, but, instead, results in formation of the glial scar composed of tightly interwoven hypertrophic reactive astrocytes. The scar plays a vital role by quickly sealing off the wound to protect the fragile nervous tissue from further erosion and to restore the blood–brain barrier, but it also prevents any meaningful regeneration across the wound gap.

Thus, in different branches of the deuterostomian clade CNS injury elicits a response from glial cells surrounding the lesioned area. The analysis of the available data suggests that the nature of this initial glial response largely determines if scarring or complete regeneration will subsequently ensue. The permissive type of the glial response and production of new neurons is associated with the persistence of radial glial cells in the adult CNS both in echinoderms and regeneration-competent vertebrates. This implies that not only are the radial glial cells a common CNS component in different deuterostomes, but also that they play similar roles in post-traumatic regeneration in these taxa. It is interesting that after ~900 million years that have passed after separation of the clades Chordata and Amulacraria [17], the radial glial cells in echinoderms and chordates still show highly stereotypical behavior in response to the neural injury. This rises a possibility that the underlying fundamental mechanisms might also be conserved and, might be present, although in an inactive or modified state, even in non-regenerating vertebrates, including human. Indeed, the echoes of these presumably ancient cellular events have been documented even in those vertebrate species, which cannot fully regenerate their CNS. For example, although lizards never completely regenerate their spinal cord after tail amputation, a narrow ependymal tube grows beyond the plane of amputation as a caudal extension of the central canal [33,37]. Similarly, although inhibitory processes eventually predominate and result in formation of a glial scar in the lesioned mammalian CNS [34], the nervous tissue still shows a clear initial regenerative response to injury, including acquisition of stem cell properties by astroglia (post-mitotic cells in the uninjured CNS) and dedifferentiation of Müller glia in the injured human brain and retina, respectively [38,39] and even formation of axonal growth cones and small amount of growth/sprouting [40]. Interestingly, although activated astrocytes (the direct developmental progeny of the radial glia) remain within their lineage in vivo, they are able to give rise to self-renewing neurospheres and generate new astrocytes, neurons, and oligodendrocytes under more permissive in vitro conditions [38]. Therefore, although examples of independent taxon-specific evolution of regenerative mechanisms exist [14], the ability to regenerate the CNS by activation of radial glia seems to be a conserved shared character of deuterostomes.

Outside the deuterostomian lineage, glial cells do not necessarily constitute the cell source for nervous system regeneration and/or adult neurogenesis. Thus, cnidarians have an elaborate nervous system composed of well-developed nerve nets and nerve rings, but completely lack cells, which can be identified as glia [2,7]. In this case, neurons are produced by multipotent interstitial cells, which also give rise to other, non-neural, cell types, such as nematocytes, gland cells, and gametes [7]. A similar scenario occurs in planarians. Although morphologically identifiable glial cell have been described in these animals [2], they are not involved in normal cell turnover or regeneration of the nervous system. Like all other differentiated cells types in the planarian body, neurons are generated from pluripotent neoblasts, the only proliferative cell type. No cell division is observed in the regenerating or fully formed planarian brain, suggesting that neoblast directly differentiate into neurons skipping the stage of dividing intermediate precursors [8]. It has also been recently suggested that in decapod crustaceans the pool of neuronal precursors is also replenished from the sources outside the nervous system, namely, from hematopoietic stem cells. These cells are though to be able to migrate into neurogenic niches to generate intermediate progenitors, which, after several cycles of cell division, differentiate into neurons [4]. Taking into account possible independent origin of glia in major metazoan clades [2], it is not surprising that non-deuterostomian taxa do not possess radial glia and seem to have evolved other mechanisms of adult neurogenesis and/or neural regeneration.

The very fact that the radial glial cells appear to be the only major cell source of CNS regeneration in echinoderms is even more interesting in view of well-known developmental plasticity of adult tissues in this phylum, where cells of different lineages can be recruited to rebuild lost or injured body parts. Thus, in some sea cucumbers, the mesothelium of the digestive tube (the outer epithelial layer of the gut wall, mesodermal in origin) is able to transcend the germ layer boundaries and give rise to the luminal epithelium (normally, endodermal in origin) in the anterior region of the regenerating gut, whereas in the posterior region the endodermal and mesodermal epithelia regenerated from their respective sources [41]. By contrast, over 93% of dividing cells in the regenerating radial nerve cord were co-labeled with the glial marker ERG1. Moreover, in our previous detailed ultrastructural analysis we also observed no contribution of external cell sources to the radial nerve regeneration [25]. Thus, the sources outside the CNS play only a negligible role, if any. This suggests the presence of certain developmental constraints, which set a limit on variations in mechanisms of neurogenesis even in basal deuterostomes, such as echinoderms.

The present study has exposed a number of issues that await further research. First, in mammals, the radial glia of the developing CNS and the astroglia of the adult nervous tissue are known to be heterogeneous in terms of their neurogenic potential and response to injury, respectively [42,43]. The radial glia of echinoderms constitute a seemingly homogeneous cell population, at least in terms of morphology. However, it is currently unknown whether all these phenotypically similar cells have equal capacity to self-renew and/or become neuronal precursors. Second, the details of the transformation of glial progenitors into neurons remain unknown for echinoderms. This is mostly due to the lack of pan-neuronal antibodies that would label all differentiating and/or mature neurons. We have the glial marker ERG1, which labels all radial glia, but this is not the case with neuronal component of the the echinoderm CNS. Whereas almost all ERG1-negative cells are morphologically identified as neurons [22], we cannot directly visualize them all at once in our immunocytochemical assays. Therefore, we are presently bound to use antibodies, which label specific neuronal subpopulations, such as GFSKLYFamide^+^, GABA^+^, Nurr1^+^, but not all neurons simultaneously. Third, the similarity of CNS regeneration at the cellular level calls for further comparative studies of molecular gene regulatory mechanisms underlying the plasticity of radial glia in echinoderms and regeneration-competent vertebrates. If many of the components of this molecular machinery are also evolutionary conserved and are present in poorly regenerating mammals, then there is certainly hope that it could be possible to convert the endogenous glial cells into neurons in the injured human central nervous system [39] using relatively minor manipulations. To this end, efforts to characterize differential gene expression associated with echinoderm CNS regeneration are well under way (Mashanov et al., in preparation). Identification of these evolutionary conserved molecular processes will be important both for fundamental developmental biology and for development of new strategies aimed at eliciting regenerative response in the injured human CNS.

## Conclusions

Our study demonstrates that echinoderms employ post-traumatic activation of radial glia to accomplish fast and efficient regeneration of their central nervous system, the process, which yields completely normal morphology and replaces the neurons lost to injury. Taken together, our data and the analysis of the available literature suggest that post-traumatic CNS regeneration via activation of radial glial cells is a conserved cellular mechanisms within the Deuterostomia, as it occurs both in echinoderms and regeneration-competent lower vertebrates. Since vestiges of this regenerative response can be seen even in poorly regenerating higher vertebrates, there is certainly hope that comparative studies can inform applied biomedical research how the phylogenetically conserved mechanisms, which might be present in a latent state, can be activated to improve neural repair in mammals, including human.

## Methods

### Animal collection and surgical procedures

Adult individuals of *Holothuria glaberrima* Selenka, 1867 (Echinodermata: Holothuroidea) were collected at low tide from rocky shores adjacent to the La Perla and Piñones districts of northeast Puerto Rico and immediately brought to the laboratory. The surgery was performed as described previously [44,45]. Briefly, autotomy of visceral organs was induced by injection of a few milliliters of 0.35 M KCl into the coelom. The eviscerated animals were left to recover for 12 h in aerated sea water at room temperature and then were anesthetized in 0.2% chlorobutanol (Sigma) dissolved in seawater until they exhibited no response to touch (typically, 10–30 min). The inner surface of the body wall was exposed through the cloaca using a blunt-end glass rod (Figure 2A – C) so as to cut the radial organs of the mid-ventral radius (including the longitudinal muscle band, radial water-vascular canal, and the radial nerve cord) at about the mid-body region (Figure 2D, E). Special care was taken to avoid damaging the outermost connective tissue layer of the body wall and the epidermis, as *H. glaberrima* poorly survives full-thickness injuries to the body wall, but readily regenerates as long as there is no direct communication between the coelom and the environment. The animals were then allowed to recover until they resumed the ability to attach themselves to the bottom or sides of the tanks and then transferred to larger tanks with fresh aerated seawater. During the first week after the surgery, the seawater was changed every day, and then weekly for the duration of the experiment.

### Histology

For general morphology, tissue samples of the uninjured and regenerating radial nerve cord were fixed in 4% paraformaldehyde with 0.1% glutaraldehyde and 0.2% picric acid in 0.01M PBS (pH 7.4, 1030 mOsm) overnight at 4°C. The samples were then rinsed in the same buffer, decalcified in 10% EDTA in 0.05 M Tris–HCl pH 7.4, dehydrated in alcohol, cleared in xylene, and embedded in paraplast (Sigma). Sections (10 μm) were collected on gelatin/chrome alum-coated slides, stained either with Mayer’s hematoxylin and eosin or with Giemsa stain (Sigma), and coverslipped in Permount (Fisher Scientific). Occasionally, cryosections (see below) were also stained with hematoxylin and eosin.

### Immunofluorescent histochemistry

For immunocytochemical analysis and cell counting assays (see below), uninjured and regenerating animals were sacrificed in groups of four. The tissue samples were fixed overnight with 4% paraformaldehyde in 0.01 M PBS at 4°C. After rinsing in the buffer, the samples were cryoprotected in graded sucrose solutions and frozen in OCT (Sakura Finetek). Cryosections (10 μm) were cut with a Leica CM1850 cryostat and collected on gelatinized slides. Immunostaining was performed as described elsewhere [21]. Briefly, slides were pre-treated with 0.5 M Triton X-100 in PBS for 30 min. Autofluorescence was quenched by incubation of the sections in 0.1 M glycine in PBS for 1 h. The slides were then incubated for 1 h in 2% normal goat serum. The antibodies used in this study, their sources and dilutions are listed in Table 1. The primary antibodies were applied overnight at 4°C. Incubation in the secondary antibodies was performed at room temperature for 1 h. The nuclei were stained with Hoechst 33342, DAPI, or propidium iodide (Sigma-Aldrich). The sections were then mounted in 25% buffered glycerol containing 0.2 M Tris–HCl (pH 8.5), 2.5% DABCO (Sigma-Aldrich), and 10% Mowiol 4–88 (Calbiochem).

**Table 1:** Antibodies used in the present study

**Antibodies used**	**Source**	**Host species**	**Dilutions**
AFRU	Dr. J. M. Grondona (University of Malaga, Spain)	Rabbit	1:1000 – 1:4000
BrdU	GenWay (20-783-71418)	Rat	1:400
ERG1	Mashanov et al. [22]	Mouse	1:1
GABA	Sigma (A2052)	Rabbit	1:1000 – 1:2000
GFSKLYFamide	Diaz-Miranda et al. [31]	Rabbit	1:500 – 1:1000
Nurr1/Nur77 (E-20)	Santa Cruz Biotechnology (SC-990)	Rabbit	1:250 – 1:1000
AMCA-conjugated AffiniPure Goat Anti-Rat IgG (H+L)	Jackson ImmunoResearch Laboratories, Inc. (112-155-003)	Goat	1:50
Cy3-conjugated AffiniPure Goat Anti-Mouse IgG (H+L)	Jackson ImmunoResearch Laboratories, Inc. (112-165-146)	Goat	1:2000
Cy3-conjugated AffiniPure Goat Anti-Rabbit IgG (H+L)	Jackson ImmunoResearch Laboratories, Inc. (112-165-144)	Goat	1:2000
FITC-conjugated Goat Anti-Mouse IgG	Biosource (AMI0408)	Goat	1:100
FITC-conjugated Goat Anti-Rabbit IgG	Biosource (ALI0408)	Goat	1:100
FITC-conjugated Goat Anti-Rat IgG (H+L)	GenWay (25-787-278232)	Goat	1:50

### Cell proliferation and cell death assays

In order to determine the percentage of dividing cells in the normal animals and at different time points of regeneration, we performed a single intracoelomic injection of 5-bromo-2-deoxyuridine (BrdU, Sigma, 50 mg/kg in 0.01 M PBS, pH 7.4). The BrdU-injected animals were returned to aquaria for 4 h and subsequently sacrificed. Tissues were processed for BrdU immunohistochemistry as described above.

Alternatively, in order to trace the progeny of BrdU incorporating cells, the regenerating animals received BrdU injections (50 mg/kg) twice a day during days 8 thru 12 post-injury. This time frame corresponded to the period of active growth and the most extensive cell division in the regenerate (see the Results section). Tissue samples were fixed 12 hours, 51 days and 133 days after the last injection and then processed for BrdU immunohistochemistry as above.

Deoxynucleotidyl transferase-mediated dUTP nick end labeling (TUNEL) was used to identify cells undergoing programmed cell death and was carried out using Fluorescein FragEL DNA Fragmentation Detection Kit (Calbiochem) following the manufacturer’s manual.

### Image acquisition and processing, cell counting, and statitical analysis

The sections were viewed and photographed with a Nikon Eclipse 600 microscope equipped with a Spot RT3 digital camera (Diagnostic Instruments, Inc). Post-aquisition image processing included composite image (overlay) generation, assembling panoramic images, brightness/contrast adjustment, multi-panel figure assembling and annotation and was carried out using the open-source software tools Fiji [46] and GIMP 2.6 [47].

Cells were counted on every third serial longitudinal section using micrographs taken with a 100× objective (see a representative example in Additional file 5: Figure S1) and the Cell Counter plugin in Fiji. Cell counting was performed in four animals per time point in the regenerate per se plus 50 μm of the adjacent stump regions (tissue that remained unaffected by the injury). The numerical data were processed using the R statistical environment ([48]. Differences between groups were analyzed with one-way ANOVA followed by Tukey’s post-hoc test. The diagrams were drawn with OpenOffice Calc software [49].

## Abbreviations

ANOVA: Analysis of variance; BrdU: 5-Bromo-2′-deoxyuridine; CNS: Central nervous system; RNC: Radial nerve cord; RS: Reissner’s substance; TUNEL: Terminal deoxynucleotidyl transferase dUTP nick end labeling.

## Competing interests

The authors declare that they have no competing interests.

## Authors’ contributions

VSM, ORZ and JEGA conceived the study and interpreted the results. ORZ and VSM carried out experimental procedures and analyzed the data. VSM drafted the manuscript. JEGA, ORZ, and VSM finalized the manuscript. All authors read and approved the final manuscript.

## Supplementary Material

Additional file 1: Table S1 Quantification of cell proliferation (through BrdU incorporation) in the normal and regenerating RNC. Click here for file

Additional file 2: Table S2 ANOVA test results for dynamics of cell proliferation. Click here for file

Additional file 3: Table S3 Quantification of programmed cell death (through TUNEL assay) in the normal and regenerating radial nerve cord. Click here for file

Additional file 4: Table S4 ANOVA test results for dynamics of programmed cell death. Click here for file

Additional file 5: Figure S1 Representative example of a micrograph used in cell counting assays. The micrograph was taken with a 100x objective. Only cells in sharp focus were counted (such as those indicated by arrows). A shows co-localization of ERG1 and BrdU labeling in the cells marked with arrows, whereas A’ and A” show these two type of labeling in separate channels. Click here for file

## References

[B1] Kettenmann H Verkhratsky A Neuroglia: the 150 years after Trends Neurosci 2008 31 653 659 10.1016/j.tins.2008.09.003 18945498

[B2] Hartline DK The evolutionary origins of glia Glia 2011 59 1215 1236 10.1002/glia.21149 21584869

[B3] Morrens J Van Den Broeck W Kempermann G Glial cells in adult neurogenesis Glia 2012 60 159 174 10.1002/glia.21247 22076934

[B4] Beltz BS Zhang Y Benton JL Sandeman DC Adult neurogenesis in the decapod crustacean brain: a hematopoietic connection? Eur J Neurosci 2011 34 870 883 10.1111/j.1460-9568.2011.07802.x 21929622 PMC3178839

[B5] Bonfanti L Rossi F Zupanc GKH Towards a comparative understanding of adult neurogenesis Eur J Neurosci 2011 34 845 846 10.1111/j.1460-9568.2011.07816.x 21929619

[B6] Ferretti P Is there a relationship between adult neurogenesis and neuron generation following injury across evolution? Eur J Neurosci 2011 34 951 962 10.1111/j.1460-9568.2011.07833.x 21929627

[B7] Galliot B Quiquand M A two-step process in the emergence of neurogenesis Eur J Neurosci 2011 34 847 862 10.1111/j.1460-9568.2011.07829.x 21929620

[B8] Umesono Y Tasaki J Nishimura K Inoue T Agata K Regeneration in an evolutionarily primitive brain–the planarian Dugesia japonica model Eur J Neurosci 2011 34 863 869 10.1111/j.1460-9568.2011.07819.x 21929621

[B9] Chernoff EAG Stocum DL Nye HLD Cameron JA Urodele spinal cord regeneration and related processes Dev Dyn 2003 226 295 307 10.1002/dvdy.10240 12557207

[B10] Sîrbulescu RF Zupanc GKH Spinal cord repair in regeneration-competent vertebrates: Adult teleost fish as a model system Brain Res Rev 2011 67 73 93 10.1016/j.brainresrev.2010.11.001 21059372

[B11] Kroehne V Freudenreich D Hans S Kaslin J Brand M Regeneration of the adult zebrafish brain from neurogenic radial glia-type progenitors Development 2011 138 4831 4841 10.1242/dev.072587 22007133

[B12] Illis LS Central nervous system regeneration does not occur Spinal Cord 2012 50 259 263 10.1038/sc.2011.132 22105462

[B13] Wicht H Lacalli T The nervous system of amphioxus: structure, development, and evolutionary significance Can J Zool 2005 83 122 150 10.1139/z04-163

[B14] Garza-Garcia AA Driscoll PC Brockes JP Evidence for the local evolution of mechanisms underlying limb regeneration in salamanders Integr Comp Biol 2010 50 528 535 10.1093/icb/icq022 21558221

[B15] Adoutte A Balavoine G Lartillot N Lespinet O Prud’homme B De Rosa R The new animal phylogeny: reliability and implications Proc Natl Acad Sci USA 2000 97 4453 4456 10.1073/pnas.97.9.4453 10781043 PMC34321

[B16] Winchell CJ Sullivan J Cameron CB Swalla BJ Mallatt J Evaluating hypotheses of deuterostome phylogeny and chordate evolution with new LSU and SSU ribosomal DNA data Mol Biol Evol 2002 19 762 776 10.1093/oxfordjournals.molbev.a004134 11961109

[B17] Blair JE Hedges SB Molecular phylogeny and divergence times of deuterostome animals Mol Biol Evol 2005 22 2275 2284 10.1093/molbev/msi225 16049193

[B18] Heinzeller T Welsch U Roth G, Wullimann M The Echinoderm Nervous System and its Phylogenetic Interpretation Brain evolution and cognition 2001 New York and Heidelberg: John Wiley & Sons, Inc. and Spektrum Akademischer Verlag 41 75

[B19] Mashanov VS Zueva OR Heinzeller T Dolmatov IY Ultrastructure of the circumoral nerve ring and the radial nerve cords in holothurians (Echinodermata) Zoomorphology 2006 125 27 38 10.1007/s00435-005-0010-9

[B20] Mashanov VS Zueva OR Heinzeller T Aschauer B Dolmatov IY Developmental origin of the adult nervous system in a holothurian: an attempt to unravel the enigma of neurogenesis in echinoderms Evol Dev 2007 9 244 256 10.1111/j.1525-142X.2007.00157.x 17501748

[B21] Mashanov VS Zueva OR Heinzeller T Aschauer B Naumann WW Grondona JM Cifuentes M Garcia-Arraras JE The central nervous system of sea cucumbers (Echinodermata: Holothuroidea) shows positive immunostaining for a chordate glial secretion Front Zool 2009 6 11 10.1186/1742-9994-6-11 19538733 PMC2705372

[B22] Mashanov VS Zueva OR Garcia-Arraras JE Organization of glial cells in the adult sea cucumber central nervous system Glia 2010 58 1581 1593 20578040 10.1002/glia.21031

[B23] Burke RD Deuterostome neuroanatomy and the body plan paradox Evol Dev 2011 13 110 115 10.1111/j.1525-142X.2010.00460.x 21210947

[B24] Viehweg J Naumann W Olsson R Secretory raidal glia in the ectoneural system of the sea star *Asterias rubens* (Echinodermata) Acta Zoologica 1998 79 119 131 10.1111/j.1463-6395.1998.tb01151.x

[B25] Mashanov VS Zueva OR Heinzeller T Regeneration of the radial nerve cord in a holothurian: A promising new model system for studying post-traumatic recovery in the adult nervous system Tissue Cell 2008 40 351 372 10.1016/j.tice.2008.03.004 18499205

[B26] San Miguel-Ruiz JE Maldonado-Soto AR García-Arrarás JE Regeneration of the radial nerve cord in the sea cucumber *Holothuria glaberrima* BMC Dev Biol 2009 9 3 10.1186/1471-213X-9-3 19126208 PMC2640377

[B27] Hyman L The Invertebrates. IV. Echinodermata. The Celomate Bilateria 1955 New York: McGraw-Hill Book Co. Inc

[B28] Viehweg J Naumann WW Radial secretory glia conserved in the postnatal vertebrate brain: a study in the rat Anat Embryol (Berl) 1996 194 355 363 8896699 10.1007/BF00198537

[B29] Lichtenfeld J Viehweg J Schutzenmeister J Naumann WW Reissner’s substance expressed as a transient pattern in vertebrate floor plate Anat Embryol (Berl) 1999 200 161 174 10.1007/s004290050270 10424874

[B30] Rodríguez EM Oksche A Hein S Rodríguez S Yulis R Comparative immunocytochemical study of the subcommissural organ Cell Tissue Res 1984 237 427 441 6435876 10.1007/BF00228427

[B31] Díaz-Miranda L Blanco RE García-Arrarás JE Localization of the heptapeptide GFSKLYFamide in the sea cucumber *Holothuria glaberrima* (Echinodermata): a light and electron microscopic study J Comp Neurol 1995 352 626 640 10.1002/cne.903520410 7722004

[B32] Ma DK Kim WR Ming G Song H Activity-dependent extrinsic regulation of adult olfactory bulb and hippocampal neurogenesis Ann N Y Acad Sci 2009 1170 664 673 10.1111/j.1749-6632.2009.04373.x 19686209 PMC2729764

[B33] McLean KE Vickaryous MK A novel amniote model of epimorphic regeneration: the leopard gecko, *Eublepharis macularius* BMC Dev Biol 2011 11 50 10.1186/1471-213X-11-50 21846350 PMC3180301

[B34] Fitch MT Silver J CNS injury, glial scars, and inflammation: Inhibitory extracellular matrices and regeneration failure Exp Neurol 2008 209 294 301 10.1016/j.expneurol.2007.05.014 17617407 PMC2268907

[B35] Sofroniew MV Molecular dissection of reactive astrogliosis and glial scar formation Trends Neurosci 2009 32 638 647 10.1016/j.tins.2009.08.002 19782411 PMC2787735

[B36] Kawano H Kimura-Kuroda J Komuta Y Yoshioka N Li HP Kawamura K Li Y Raisman G Role of the lesion scar in the response to damage and repair of the central nervous system Cell Tissue Res 2012 349 169 180 10.1007/s00441-012-1336-5 22362507 PMC3375417

[B37] Egar M Simpson SB Singer M The growth and differentiation of the regenerating spinal cord of the lizard, *Anolis carolinensis* J Morphol 1970 131 131 151 10.1002/jmor.1051310202 5425076

[B38] Buffo A Rite I Tripathi P Lepier A Colak D Horn A Mori T Götz M Origin and progeny of reactive gliosis: A source of multipotent cells in the injured brain Proc Natl Acad Sci USA 2008 105 3581 3586 10.1073/pnas.0709002105 18299565 PMC2265175

[B39] Berninger B Making neurons from mature glia: a far-fetched dream? Neuropharmacology 2010 58 894 902 10.1016/j.neuropharm.2009.11.004 19931285

[B40] Kerschensteiner M Schwab ME Lichtman JW Misgeld T In vivo imaging of axonal degeneration and regeneration in the injured spinal cord Nat Med 2005 11 572 577 10.1038/nm1229 15821747

[B41] Mashanov VS Dolmatov IY Heinzeller T Transdifferentiation in holothurian gut regeneration Biol Bull 2005 209 184 193 10.2307/3593108 16382166

[B42] Pinto L Götz M Radial glial cell heterogeneity–the source of diverse progeny in the CNS Prog Neurobiol 2007 83 2 23 10.1016/j.pneurobio.2007.02.010 17580100

[B43] White RE McTigue DM Jakeman LB Regional heterogeneity in astrocyte responses following contusive spinal cord injury in mice J Comp Neurol 2010 518 1370 1390 20151365 10.1002/cne.22282 PMC2867111

[B44] San Miguel-Ruiz JE García-Arrarás JE Common cellular events occur during wound healing and organ regeneration in the sea cucumber *Holothuria glaberrima* BMC Dev Biol 2007 7 115 10.1186/1471-213X-7-115 17945004 PMC2176065

[B45] Mashanov VS Zueva OR García-Arrarás JE Posttraumatic regeneration involves differential expression of long terminal repeat (LTR) retrotransposons Dev Dyn 2012 241 1625 1636 10.1002/dvdy.23844 22911496

[B46] http://fiji.sc.

[B47] http://www.gimp.org.

[B48] http://www.r-project.org.

[B49] https://www.openoffice.org.

