# Supplementary material for: Radial glial cells play a key role in echinoderm neural regeneration
Source: BMC Biol. 2013 Apr 18;11:49. doi: 10.1186/1741-7007-11-49 (PMC3652774; doi:10.1186/1741-7007-11-49)
Supplement: Additional file 1: Table S1 — Quantification of cell proliferation (through BrdU incorporation) in the normal and regenerating RNC. [file 1741-7007-11-49-S1.pdf]

Supplementary Table 1. Quantification of cell proliferation (through BrdU incorporation) in the normal and regenerating RNC

(A) Phenotype ratio: (all BrdU+ cells) ÷ (total cell number), Mean ±SE, %

	RNC region		
	Ectoneural	Hyponeural	RNC as a whole
Normal animals	0.45±0.10	0.77±0.11	0.53±0.07
Early post-injury phase	0.14±0.07	0.08±0.08	0.14±0.07
Late post-injury phase	3.34±0.76	1.96±0.26	2.98±0.62
Growth phase	5.28±0.98	5.86±1.40	5.34±0.94
Late regenerate	1.74±0.50	2.58±1.13	1.87±0.59

(B) Phenotype ratio: (ERG1+ BrdU+ cells) ÷ (all BrdU+ cells), Mean ±SE, %

	RNC region		
	Ectoneural	Hyponeural	RNC as a whole
Normal animals	90.36±6.74%	96.43±0.07	91.10±4.72
Early post-injury phase	100.00±0.00%	‡	100.00±0.00
Late post-injury phase	96.30±0.37	100.00±0.00	97.00±0.34
Growth phase	97.58±0.63	99.00±1.00	97.78±0.66
Late regenerate	93.77±2.11	98.44±1.56	94.96±1.62

(C) Phenotype ratio: (ERG1+ BrdU+ cells) ÷ (total ERG1+ cell number), Mean ±SE, %

	RNC region		
	Ectoneural	Hyponeural	RNC as a whole
Normal animals	0.59±0.13	0.98±0.13	0.70±0.10
Early post-injury phase	0.20±0.09	0.05±0.05	0.19±0.09
Late post-injury phase	5.10±1.22	2.57±0.30	4.32±0.92
Growth phase	7.07±1.18	6.77±1.59	6.85±1.05
Late regenerate	2.79±0.83	3.34±1.31	2.89±0.92

‡ indicates that the value is missing because BrdU-incorporating cells were absent in three of the four animals. The the ratio of the number of BrdU+ ERG1+ cells divided by the total number of BrdU+ cells is impossible to define in these animals (division of zero by zero). Therefore, neither mean value nor standard error were calculated. In the fourth animal, there were only two BrdU+ cells in the hyponeural neuroepithelium, one of them was BrdU+ ERG1+, whereas the other was BrdU+ ERG1-
