# Supplementary material for: Radial glial cells play a key role in echinoderm neural regeneration
Source: BMC Biol. 2013 Apr 18;11:49. doi: 10.1186/1741-7007-11-49 (PMC3652774; doi:10.1186/1741-7007-11-49)
Supplement: Additional file 2: Table S2 — ANOVA test results for dynamics of cell proliferation. [file 1741-7007-11-49-S2.pdf]

Supplementary Table 2. ANOVA test results for dynamics of cell proliferation

**(A) Phenotype ratio: (all BrdU+ cells) ÷ (total cell number)**			
	RNC region		
	Ectoneural	Hyponeural	RNC as a whole
F(4, 15)	12.59	7.54	13.51
P	1.09E-004	1.55E-003	7.32E-005

**(B) Phenotype ratio: (ERG1+ BrdU+ cells) ÷ (all BrdU+ cells)**			
	RNC region		
	Ectoneural	Hyponeural	RNC as a whole
F(4, 15)	1.35	0.56	2.22
P	2.96E-001	6.52E-001	1.15E-001

**(C) Phenotype ratio: (ERG1+ BrdU+ cells) ÷ (total ERG1+ cell number)**			
	RNC region		
	Ectoneural	Hyponeural	RNC as a whole
F(4, 15)	11.90	7.75	13.34
P	1.49E-004	1.36E-003	7.87E-005
