# Supplementary material for: Radial glial cells play a key role in echinoderm neural regeneration
Source: BMC Biol. 2013 Apr 18;11:49. doi: 10.1186/1741-7007-11-49 (PMC3652774; doi:10.1186/1741-7007-11-49)
Supplement: Additional file 3: Table S3 — Quantification of programmed cell death (through TUNEL assay) in the normal and regenerating radial nerve cord. [file 1741-7007-11-49-S3.pdf]

Supplementary Table 3. Quantification of programmed cell death (through TUNEL assay) in the normal and regenerating radial nerve cord

(A) Phenotype ratio: (all TUNEL+ cells) ÷ (total cell number), Mean ±SE, %			
	RNC region		
	Ectoneural	Hyponeural	RNC as a whole
Normal animals	0.21±0.04	0.20±0.08	0.21±0.04
Early post-injury phase	4.92±0.94	2.86±0.77	4.34±0.88
Late post-injury phase	1.35±0.37	2.13±0.26	1.52±0.33
Growth phase	1.94±0.30	1.53±0.57	1.84±0.37
Late regenerate	0.54±0.09	1.44±0.23	0.73±0.07

(B) Phenotype ratio: (ERG1+ TUNEL+ cells) ÷ (total TUNEL+ cell number), Mean ±SE, %			
	RNC region		
	Ectoneural	Hyponeural	RNC as a whole
Normal animals	45.00±17.92	37.50±23.94	50.60±15.28
Early post-injury phase	36.83±11.04	35.68±5.64	34.76±7.38
Late post-injury phase	53.35±5.04	67.98±9.85	57.62±5.80
Growth phase	60.61±5.95	86.61±7.77	64.41±5.46
Late regenerate	47.04±18.44	61.32±12.72	53.10±10.01

(C) Phenotype ratio: (ERG1+ TUNEL+ cells) ÷ (total ERG1+ cell number), Mean ±SE, %			
	RNC region		
	Ectoneural	Hyponeural	RNC as a whole
Normal animals	0.16±0.07	0.17±0.12	0.16±0.07
Early post-injury phase	2.78±0.79	1.32±0.25	2.09±0.62
Late post-injury phase	1.44±0.38	2.28±0.42	1.62±0.34
Growth phase	1.75±0.31	1.64±0.58	1.71±0.35
Late regenerate	0.37±0.09	1.17±0.21	0.60±0.08
