# Supplementary material for: Radial glial cells play a key role in echinoderm neural regeneration
Source: BMC Biol. 2013 Apr 18;11:49. doi: 10.1186/1741-7007-11-49 (PMC3652774; doi:10.1186/1741-7007-11-49)
Supplement: Additional file 4: Table S4 — ANOVA test results for dynamics of programmed cell death. [file 1741-7007-11-49-S4.pdf]

Supplementary Table 4. ANOVA test results for dynamics of programmed cell death

**(A) Phenotype ratio: (all TUNEL+ cells) ÷ (total cell number)**			
	RNC region		
	Ectoneural	Hyponeural	RNC as a whole
F(4, 15)	15.62	4.58	12.51
P	3.16E-005	1.29E-002	1.13E-004

**(B) Phenotype ratio: (ERG1+ TUNEL+ cells) ÷ (total TUNEL+ cell number)**			
	RNC region		
	Ectoneural	Hyponeural	RNC as a whole
F(4, 15)	0.47	2.50	1.34
P	7.54E-001	8.67E-002	0.30

**(C) Phenotype ratio: (ERG1+ TUNEL+ cells) ÷ (total ERG1+ cell number)**			
	RNC region		
	Ectoneural	Hyponeural	RNC as a whole
F(4, 15)	6.50	4.64	5.21
P	3.07E-003	1.23E-002	7.81E-003
