# Supplementary figures and images for: Radial glial cells play a key role in echinoderm neural regeneration
Source: BMC Biol. 2013 Apr 18;11:49. doi: 10.1186/1741-7007-11-49 (PMC3652774; doi:10.1186/1741-7007-11-49)

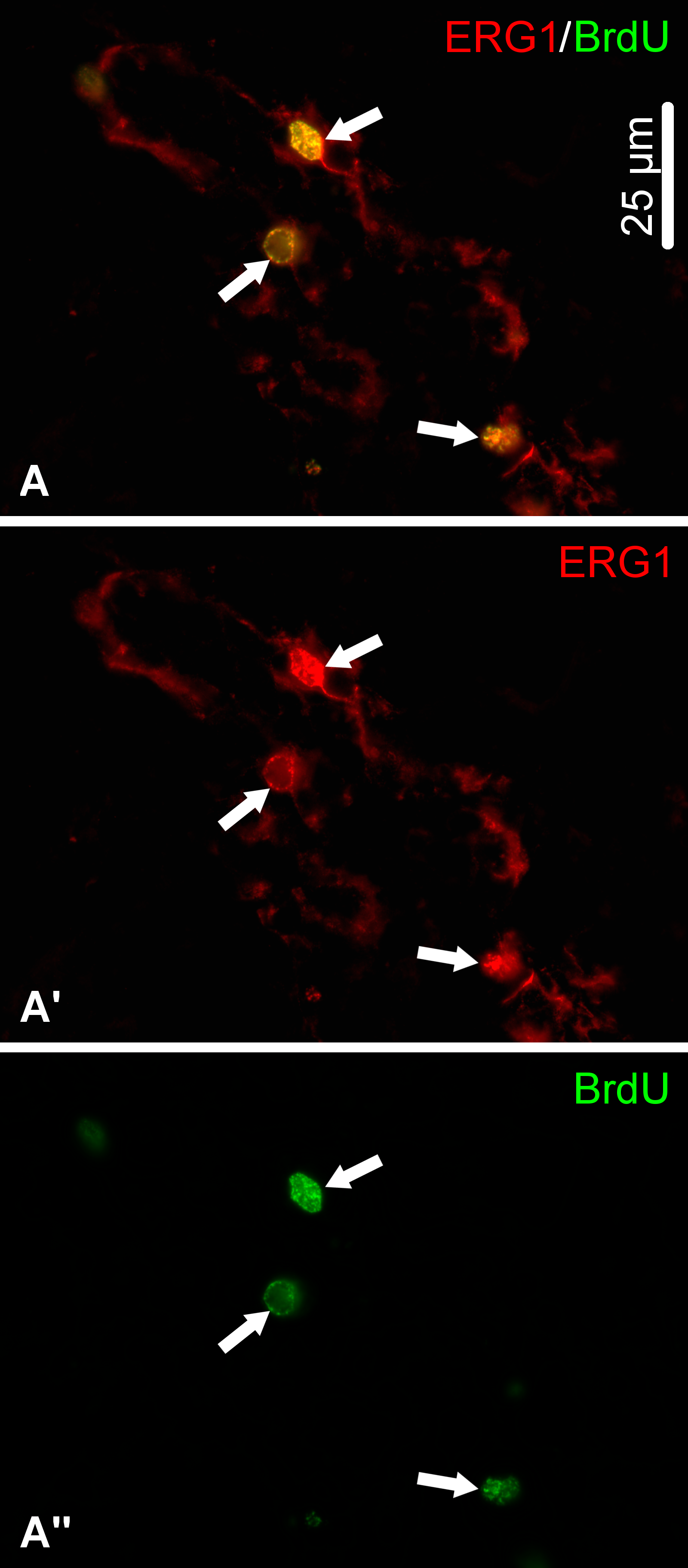

Supplement: Additional file 5: Figure S1 — Representative example of a micrograph used in cell counting assays. The micrograph was taken with a 100x objective. Only cells in sharp focus were counted (such as those indicated by arrows). A shows co-localization of ERG1 and BrdU labeling in the cells marked with arrows, whereas A’ and A” show these two type of labeling in separate channels. [file 1741-7007-11-49-S5.tiff]
